# Instantaneous mental workload assessment using time–frequency analysis and semi-supervised learning

**DOI:** 10.1007/s11571-020-09589-3

**Published:** 2020-05-12

**Authors:** Jianhua Zhang, Jianrong Li, Rubin Wang

**Affiliations:** 1 AI Lab, Department of Computer Science, Oslo Metropolitan University, 0166 Oslo, Norway; 2 School of Information Science and Engineering, East China University of Science and Technology, Shanghai, 200237 China; 3 Institute for Cognitive Neurodynamics, School of Sciences, East China University of Science and Technology, Shanghai, 200237 China

**Keywords:** Mental workload, Operator functional state, Physiological signals, Time–frequency analysis, Semi-supervised learning

## Abstract

The real-time assessment of mental workload (MWL) is critical for development of intelligent human–machine cooperative systems in various safety–critical applications. Although data-driven machine learning (ML) approach has shown promise in MWL recognition, there is still difficulty in acquiring a sufficient number of labeled data to train the ML models. This paper proposes a semi-supervised extreme learning machine (SS-ELM) algorithm for MWL pattern classification requiring only a small number of labeled data. The measured data analysis results show that the proposed SS-ELM paradigm can effectively improve the accuracy and efficiency of MWL classification and thus provide a competitive ML approach to utilizing a large number of unlabeled data which are available in many real-world applications.

## Introduction

Automation, automatic control system, and artificial intelligence (AI) techniques have been widely applied to various fields, but there is still a long way for the current automation and AI technologies to achieve fully-automated control for many real-world complex and uncertain systems. In this connection, human–machine systems (HMS) are still ubiquitous in most safety–critical application domains (Lal and Craig 2001). Compared with machines, human operators are more susceptible to the impact of external disturbances or their own psychophysiological fluctuations (Bobko et al. 1998). Therefore, it is not surprising that human factors play a significant part in the performance of HMS. In recent years, researchers from different disciplines have investigated how to maintain the optimum Operator Functional State (OFS) to ensure the successful performance of the tasks in the HMS context (Hollender et al. 2010).

The operator’s mental workload (MWL) is an essential dimension of the multi-dimensional construct of OFS. The MWL can be considered as a quantitative variable for measurement of mental status of human operator, which reflects the mental demand for operators to accomplish the tasks (Cain 2007). For operators, unduely high or low psychological  or mental load is detrimental to the performance of HMS. In order to mitigate this problem, researchers proposed Adaptive Automation (AA) strategy. The AA system can adaptively allocate the tasks between operators and the machines based on the estimated levels of operators’ MWL. MWL measurement approaches can be roughly divided into three categories (Mahfouf et al. 2007): (1) subjective assessment; (2) task performance measures; and (3) physiological data based assessment. Compared with the former two approaches, the last approach is featured by continuous on-line measurement. ElectroEncephaloGram (EEG), ElectroCardioGram (ECG) and ElectroOculoGram (EOG) have been widely used for MWL recognition (Zhang et al. 2013a, b, 2016; Wang et al. 2018; Zeng et al. 2018; Lamti et al. 2019; Mora-Sánchez et al. 2020). In this paper, we evaluate the operator MWL by using physiological signals and a semi-supervised learning technique in order to enhance the accuracy and efficiency of high-risk MWL detection.

The rest of this paper is organized as follows. “Literature review” section reviews briefly the state of the art of MWL detection and semi-supervised learning research. In “Semi-supervised learning method” section, we describe the Semi-Supervised Extreme Learning Machine (SS-ELM) method. “Feature extraction algorithms” section develops two physiological feature extraction algorithms. The data acquisition experiment and data processing method are described in “Data acquisition and data preprocessing” section. “Classification results and discussion” section presents the MWL classification results of the proposed Semi-Supervised Learning (SSL) technique, along with some meaningful discussions. Finally “Conclusion” section concludes the paper.

## Literature review

### Real-time MWL recognition

Under high MWL, the operator may ignore some important information, which would have a negative even catastrophic impact on the normal operation of the HMS (Wilson and Russell 2003b, 2007). Therefore, we have to study how to maintain the optimal OFS in safety–critical HMSs in public transport (driving, rail, shipping, aviation, and aerospace), nuclear engineering, and other similar fields. OFS can be defined as the variable capacity of the operator for effective task performance in complex and uncertain task environment as well as the limitations imposed by cognitive and physiological processes (Hockey 2003). The MWL, which is closely correlated to the transient cognitive demand, is a crucial dimension of OFS (Hockey 1997).

Real-time risky MWL detection system relates the operator’s real-time operational state to load level. There are three types of measures for MWL detection (Cannon et al. 2012): subjective rating, task performance measures, and physiological markers. MWL is traditionally evaluated by the operator’s subjective rating usually composed of several different rating scales. The rating scales are usually filled out during or after the experiment, then their statistics (e.g., mean) are used to derive a single WML index. One of the most typical subjective rating is the NASA-TLX questionnaire, which consists of six indicators, including psychological demand, physical demand, time demand, effort, and frustration (Hart and Staveland 1988). Among them, the task load index (TLI) is the weighted average of the six indicators. Another traditional method is to use performance measures. The two conventional methods are usually only feasible for offline data analysis, but not suitable for online data analysis. For example, for subjective ratings, there is no continuity in subjective scoring in online situation. For performance measures, performance data is not easily available for some systems, e.g., nuclear power plant. The neurophysiological signals can be used to realize online real-time MWL assessment mainly for the following reasons: (1) the physiological signals can reflect the operator’s inherent state; and (2) the operator state can be monitored in real-time without disturbing the task performance (Swangnetr and Kaber 2013). Therefore, we use heterogeneous (multi-source) physiological signals (particularly EEG, ECG and EOG because of their high temporal resolution and ease of measurement) to evaluate MWL in this work.

Many researchers used physiological measures to analyze OFS. In Hankins and Wilson (1998), heart rate, EOG and EEG signals were used to assess the psychological load of the pilot during flight. In Zhang et al. (2008), the MWL changes were quantitatively analyzed by heart rate variability. In Dussault et al. (2004), Dussault, Jouanin and Guezennec studied the variations in EEG and ECG under different tasks. In Fournier et al. (1999), the MWL was evaluated by using physiological signals measured under complicated task environment. In Wilson and Fisher (1991), the difficulty level of the flight phase was determined by using ECG and blink rate. In Wilson and Russell (2003a), physiological data was used for OFS analysis in an air traffic control system. Dussault et al. (2005) studied the changes of EEG and ECG as operator’s mental load and vigilance change. In Gevins et al. (1998), the EEG pattern recognition method was used to monitor the operator workload in computer tasks. Gevins et al. (1997) analyzed the effects of task difficulty and processing type on the high-resolution EEG mapping of the cerebral cortex associated with working memory. Freeman et al. (1999) used three EEG indicators under a visual tracking task to evaluate an AA system. Wilson and Russell (1999) used physiological and performance features to classify the OFS using artificial neural networks. According to the above literature overview, we can find that most MWL measurement methods proposed in literature are based on the idea of supervised learning. In real-world applied (operational) environment, the manual data labeling can be a tedious and time-consuming task which is also prone to mistakes/errors. Correct labeling of massive data requires taking into account simultaneously the subjective evaluation, performance data and other factors. Under naturalistic operational environment, the operator voluntarily decides which tasks to perform at any time, and the manual labeling of data becomes infeasible. On the other hand, it is relatively easier to collect large amounts of unlabeled data. In order to make full use of these low-cost unlabeled data, SSL is applied for model construction because it is capable of extracting useful information from both labeled and unlabeled data. The main aim of this study is to investigate the effectiveness of SSL for improving MWL recognition accuracy using both labelled and unlabeled physiological data.

### Semi-supervised learning (SSL)

In recent years, SSL has drawn intensive attention from researchers in the ML field. In the semi-supervised classification, a small number of data (with known target labels) and a large number of unlabeled data are used to construct the decision model. The SSL has wide-ranging practical applicability due to the fact that it is too expensive or time-consuming to label large amounts of data, but vast quantities of unlabeled data are available in a variety of real-world problems, e.g., online texts, protein sequences, or images.

The SSL research can be traced back to the 1970s when self-training, transductive learning, generative model and other learning methods were proposed. Self-training is the first method of applying unlabeled samples to learning problem (McClosky et al. 2006). Based on predictions, unlabeled data with high level of confidence in belonging to a certain class is converted into labeled data to further refine the model. Over the past few years, SSL algorithms based on the density separation or graph has gained much momentum. There are two state of the art SSL methods: discriminative method and graph-based method. A major class of SSL methods, so-called low-density separation method, attempts to place boundaries in regions with few data points (labeled or unlabeled), so-called low-density regions. Discriminative methods, such as transductive support vector machine (TSVM) (Joachims 1999) and semi-supervised support vector machine (S^3^VM) (Chapelle et al. 2006), use the maximum interval algorithm to simultaneously train the labeled samples and the decision boundary of the unlabeled data to pass through the low-density areas and make maximum distance from the learning superclass to the nearest sample, which relies on cluster assumption (i.e., the data tend to form discrete clusters, and points in the same cluster are more likely to share a label). One of the most commonly used algorithms is the transductive support vector machine (TSVM). Whereas SVMs for supervised learning seek a decision boundary with maximal margin over the labeled data, the goal of TSVM is a labeling of the unlabeled data such that the decision boundary has maximal margin over all of the data. On the other hand, the graph-based approach relies on manifold assumption (i.e., the data lie approximately on a manifold of much lower dimension than the input space). Generally, graph-based methods first construct a weight graph capturing the local structure and then learn a decision boundary that preserves the combination of local structures. In other words, data points that are locally close to each other should be predicted to have the same class label. Graph-based methods for SSL use a graph representation of the data, with a node for each labeled and unlabeled example. The graph may be constructed using domain knowledge or similarity of examples. Two common methods are to connect each data point to its k nearest neighbors or to examples within some distance ε. Within the framework of manifold regularization, the graph serves as a proxy for the manifold. A term is added to the standard Tikhonov regularization problem to enforce smoothness of the solution relative to the manifold (in the intrinsic space of the problem) as well as relative to the ambient input space. The Laplacian can also be used to extend the supervised learning algorithms, e.g., regularized least squares and SVM, to their semi-supervised versions. The graph-based SSL achieved satisfactory performance in text and image classification (Gómez-Chova et al. 2007; Garla et al. 2013). Because the graph-based SSL model can adequately characterize the physiological signals, we use it in this paper.

## Semi-supervised learning method

**Fig. 1:**
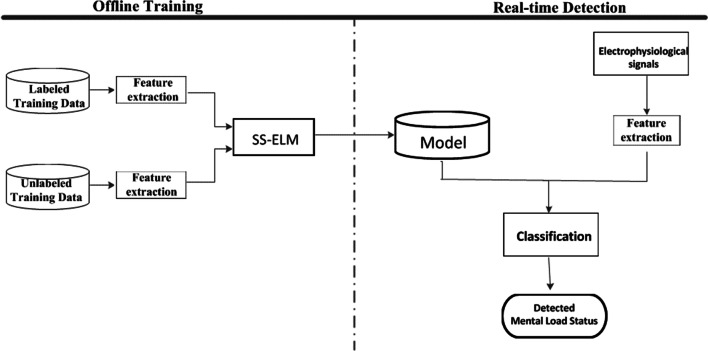
Block diagram of SSL-based MWL detection scheme

Operator’s MWL recognition is a multi-classification problem, where each MWL state is classified into three classes, i.e., baseline, low and high. Figure 1 shows the flowchart of risky MWL detection algorithm, which comprises offline training and online (real-time) detection. There are two types of data that can be used for training, i.e., labeled and unlabeled data.

In SSL setting, we usually have few labeled data and large amount of unlabeled data. We have the dataset $X = \{ (\varvec{x}_{1} ,y_{1} ), \ldots ,(\varvec{x}_{l} ,y_{l} ),\varvec{x}_{l + 1} , \ldots ,\varvec{x}_{n} \}$, in which the first *l* samples are labeled data in the training set and the remaining (*n* − *l*) samples are unlabeled data, *l* and *n* are the number of the labeled and entire data, respectively, $\varvec{x}_{i} \in {\mathbb{R}}^{D}$ represents the input measures and ${\mathbf{y}}_{i} \in {\mathbb{R}}^{c}$ is a *c*-dimensional binary vector with only one entry (corresponding to the class that **x**_*i*_ belongs to) equal to one for multi-class classification tasks, and *D* and *c*are the dimensionality of input and output, respectively.

### Manifold regularization

SSL is built on two assumptions: (1) both the labeled data *X*_*l*_ and the unlabeled data *X*_*n*−*l*_ are drawn from the same marginal distribution P_*X*_ and (2) if two points **x**_*i*_ and **x**_*j*_ are close to each other, the conditional probabilities $P\left( {{\mathbf{y}}\left| {{\mathbf{x}}_{i} } \right.} \right)$ and $P\left( {{\mathbf{y}}\left| {{\mathbf{x}}_{j} } \right.} \right)$ should be similar as well. The latter assumption is known as smoothness assumption in ML. The purpose of introducing regularization in ML is to increase the smoothness of the model so as to prevent the model from over-fitting. The manifold regularization framework is based on the assumption that high-dimensional training data (both labeled and unlabeled) from each class lie on (are embedded in) a low-dimensional manifold, and the optimal decision boundary is smooth w.r.t. the manifold. The low-dimensional manifold is represented as a collection of many small neighborhoods, where high-dimensional data points sharing the same label are close to each other.

To force the decision boundary to be ‘smooth’ to the manifold (smoothness assumption on the data/model), the manifold regularization framework proposes to minimize the loss function:

1 $$\hat{L}_{c} = \frac{1}{2}\sum\limits_{i,j} {q_{ij} \left\| {{\hat{\mathbf{y}}}_{i} - {\hat{\mathbf{y}}}_{j} } \right\|}^{2}$$

where $\left\| \cdot \right\|$ denotes the Euclidean norm, ${\hat{\mathbf{y}}}_{i} {\kern 1pt} {\kern 1pt} {\kern 1pt} \text{and}{\kern 1pt} {\hat{\mathbf{y}}}_{j}$ are the predicted output w.r.t. samples **x**_*i*_ and **x**_*j*_, respectively, and *q*_*ij*_ is the pair-wise similarity coefficient between **x**_*i*_ and **x**_*j*_. Note that the similarity matrix $Q = \left[ {q_{ij} } \right]$ is usually sparse since we only place a nonzero weight between two samples **x**_*i*_ and **x**_*j*_ if they are close, e.g., **x**_*i*_ is among the *k* nearest neighbors of **x**_*j*_ or **x**_*j*_ is among the *k* nearest neighbors of **x**_*i*_. The nonzero weights are usually calculated using Gaussian function $\exp \left[ { - {{\left\| {{\mathbf{x}}_{i} - {\mathbf{x}}_{j} } \right\|^{2} } \mathord{\left/ {\vphantom {{\left\| {{\mathbf{x}}_{i} - {\mathbf{x}}_{j} } \right\|^{2} } {2\sigma^{2} }}} \right. \kern-0pt} {2\sigma^{2} }}} \right]$ or simply set to one.

Intuitively, (1) penalizes large variation in the predicted class labels w.r.t. two nearby data points **x**_*i*_ and **x**_*j*_ (when **x** has a small change).

Equation (1) can be compactly expressed in matrix form:

2 $$\hat{L}_{c} = {\text{Tr}}\left( {\hat{Y}^{T} L\hat{Y}} \right){\kern 1pt}$$

where Tr(·) denotes the trace of a matrix, $\hat{Y} = [{\hat{\mathbf{y}}}_{1} ,{\hat{\mathbf{y}}}_{2} , \ldots ,{\hat{\mathbf{y}}}_{l} , \ldots ,{\hat{\mathbf{y}}}_{n} ]^{T}$ is the predicted output matrix of ELM, $L = D - Q$ is the *graph Laplacian*, $Q = [q_{ij} ]$ is the similarity matrix (Chapelle et al. 2006; Zhu 2017), and *D* is a diagonal matrix with $d_{ii} = \sum\nolimits_{j = 1}^{n} {q_{ij} }$.

Instead of using *L* directly, based on some a priori knowledge, *L* can be normalized by $D^{{ ( { - 1/2)}}} LD^{( - 1/2)}$ or replaced by *L*^*p*^ (*p* is an integer).

### Extreme learning machine (ELM)

Proposed by Huang et al. (2006, 2012), Extreme Learning Machine (ELM) is a unified learning scheme for *generalized* Single-hidden Layer Feedforward Neural network model (SLFNs). Compared with the traditional neural networks, ELM is faster with guaranteed learning precision.

ELM aims to learn a decision rule or an approximation function based on the training data. In general, the training of ELM consists of two stages. In ELM theory (Huang et al. 2006), the parameters of the hidden activation functions can be randomly generated and fixed according to any continuous probability distribution, e.g., the uniform distribution on (− 1, 1). This makes the ELM distinct from the traditional feedforward neural networks and SVMs. During the training process, we only need to optimize the output weight matrix *W* that connects the hidden neurons and the output nodes. By doing so, training ELM is equivalent to solving a regularized LS problem, which is much more efficient than SVM training or BP learning algorithm.

The purpose of stage 1 is to construct the hidden layer using a fixed number of randomly generated mapping neurons, which can be any nonlinear piecewise continuous function, such as the sigmoidal function. In stage 1, *L* hidden neurons, which map the data from the input space to a *L*-dimensional feature space, are generated at random.

Given a sample data ${\mathbf{x}}_{i} \in {\mathbb{R}}^{D}$, the outputs of the network with *L* hidden neurons and a sigmoidal activation function of **x**_*i*_ can be expressed by:

3 $${\mathbf{y}}_{i} = \sum\limits_{j = 1}^{L} {h_{j} ({\mathbf{x}}_{i} ;{\varvec{\uptheta}})} {\mathbf{w}}_{j} = {\mathbf{h}}({\mathbf{x}}_{i} ;{\varvec{\uptheta}})W{\kern 1pt} {\kern 1pt} ,\quad i = 1,2, \ldots ,l.{\kern 1pt} {\kern 1pt}$$

where ${\varvec{\uptheta}} = \{ {\mathbf{a}}_{j} ,b_{j} \}$ is the parameter set of the sigmoidal activation function:

4 $$h_{j} ({\mathbf{x}}_{i} ;{\varvec{\uptheta}}) = \frac{1}{{1 + \exp \left[ { - ({\mathbf{a}}_{j}^{T} {\mathbf{x}}_{i} + b_{j} )} \right]}}{\kern 1pt} {\kern 1pt} {\kern 1pt} ,\quad j = 1, \ldots ,L$$

$h_{j} ({\mathbf{x}}_{i} )$ is the output of the *j*th hidden neurons in response to the input sample **x**_*i*_, $W \in {\mathbb{R}}^{L \times c}$ is the output weight matrix that connects the hidden layer with the output layer, ${\mathbf{h}}({\mathbf{x}}_{i} ) = [h_{1} ({\mathbf{x}}_{i} ),h_{2} ({\mathbf{x}}_{i} ), \ldots ,h_{L} ({\mathbf{x}}_{i} )] \in {\mathbb{R}}^{1 \times L}$ is the output vector of the hidden layer w.r.t. **x**_*i*_, and ${\mathbf{y}} \in {\mathbb{R}}^{1 \times c}$ is the output vector of ELM and the predicted class corresponds to the label of the entry with largest output value. This enables ELM and SS-ELM to be naturally suited to multiclass classification problems.

In stage 2, ELM aims to solve the output weights by minimizing the squared sum of the prediction errors:

5 $$\begin{aligned} \mathop {\hbox{min} }\limits_{{W \in {\mathbb{R}}^{L \times c} }} {\kern 1pt} {\kern 1pt} {\kern 1pt} \frac{1}{2}\left\| W \right\|{\kern 1pt}^{2} + \frac{C}{2}\sum\limits_{i = 1}^{l} {\left\| {\xi_{i} } \right\|^{2} } \hfill \\ {\kern 1pt} {\kern 1pt} {\kern 1pt} {\kern 1pt} {\kern 1pt} {\kern 1pt} {\kern 1pt} {\kern 1pt} {\kern 1pt} {\kern 1pt} {\text{s}} . {\text{t}} .{\kern 1pt} {\kern 1pt} {\kern 1pt} {\kern 1pt} {\kern 1pt} {\kern 1pt} {\kern 1pt} {\kern 1pt} {\kern 1pt} {\kern 1pt} {\mathbf{h}}\left( {{\mathbf{x}}_{i} } \right)W = {\mathbf{y}}_{i}^{T} - \xi_{i}^{T} ,{\kern 1pt} {\kern 1pt} {\kern 1pt} {\kern 1pt} {\kern 1pt} {\kern 1pt} {\kern 1pt} {\kern 1pt} {\kern 1pt} {\kern 1pt} {\kern 1pt} {\kern 1pt} {\kern 1pt} {\kern 1pt} {\kern 1pt} {\kern 1pt} {\kern 1pt} {\kern 1pt} i = 1,2, \ldots ,l{\kern 1pt} \hfill \\ \end{aligned}$$

where the first term is a regularization term used to prevent over-fitting, *C* is a penalty factor on the training errors, and $\xi_{i} \in {\mathbb{R}}^{c}$ is the error vector of the *i*th training sample.

### Semi-supervised extreme learning machine (SS-ELM)

The SS-ELM is a semi-supervised learning algorithm based on ELM theory and manifold regularization framework, which can take advantage of the unlabeled data to improve the classification accuracy when labeled data are scarce (Huang et al. 2014). It determines the output weights by minimizing the squared sum of the empirical training error of labeled data, the norm of the output weights, as well as the manifold regularization term based on both labeled and unlabeled data.

By modifying the ELM formulation (5), we have the SS-ELM formulation:

6 $$\begin{aligned} \mathop {\hbox{min} }\limits_{{W \in {\mathbb{R}}^{L \times c} }} \frac{1}{2}\left\| W \right\|^{2} + \frac{1}{2}\sum\limits_{i = 1}^{l} {C_{{{\mathbf{y}}_{i} }} \left\| {\xi_{i} } \right\|^{2} + \frac{\lambda }{2}} {\text{Tr}}(Y^{T} LY) \hfill \\ {\mathbf{h}}({\mathbf{x}}_{i} )W\varvec{ = }{\mathbf{y}}_{i}^{T} - \xi_{i}^{T} ,\quad i = 1,2, \ldots ,l \hfill \\ {\mathbf{y}}_{i} = {\mathbf{h}}\left( {{\mathbf{x}}_{i} } \right)W,\quad i = 1,2, \ldots ,n{\kern 1pt} {\kern 1pt} {\kern 1pt} \hfill \\ \end{aligned}$$

where *λ* is a tradeoff parameter, $C_{{{\mathbf{y}}_{i} }}$ is a penalty factor for training error of data from class *y*_*i*_, $L \in {\mathbb{R}}^{n \times n}$ is the graph Laplacian built from both labeled and unlabeled data, and $Y \in {\mathbb{R}}^{n \times c}$ is the output matrix of the network with its *i*th row equal to **y**_*i*_.

Note that similar to the weighted ELM (W-ELM) algorithm, here we assign different penalty factor $C_{{{\mathbf{y}}_{i} }}$ to the prediction errors w.r.t. samples from different classes because when the data is unbalanced, i.e., some classes have significantly more samples than other classes, traditional ELM tend to fit the majority classes well, but fits minority classes poorly. This usually results in poor generalization to the testing set. Therefore, in order to cope with the possibly imbalanced classification problem, we reweigh examples from different classes. Suppose that **x**_*i*_ belongs to class **y**_*i*_ which has $N_{{{\mathbf{y}}_{i} }}$ training samples, then we assign $\xi_{i}$ with a penalty of $C_{{{\mathbf{y}}_{i} }} = \frac{{C_{0} }}{{N_{{{\mathbf{y}}_{i} }} }}$, where *C*_0_ is a user-defined parameter as in traditional ELM and $N_{{{\mathbf{y}}_{i} }}$ is the number of training samples in the class **y**_*i*_. In this way, the samples from the dominant classes will not be overfitted by the algorithm and the samples from a class with less samples will not be ignored.

Substituting the constraints into the objective function yields the new formulation in matrix form:

7 $$\mathop {\hbox{min} }\limits_{{W \in {\mathbb{R}}^{{L \times {\text{c}}}} }} \left[ {\frac{1}{2}\parallel W\parallel^{2} + \frac{1}{2}\parallel C^{{\frac{1}{2}}} (\tilde{Y} - HW)\parallel^{2} + \frac{\lambda }{2}{\text{Tr}}(W^{T} H^{T} LHW)} \right]$$

where $H = [{\mathbf{h}}({\mathbf{x}}_{1} )^{T} ,{\mathbf{h}}({\mathbf{x}}_{2} )^{T} , \ldots ,{\mathbf{h}}({\mathbf{x}}_{l} )^{T} ]^{T} \in {\mathbb{R}}^{l \times L} ,\tilde{Y} \in {\mathbb{R}}^{n \times c}$ is the augmented training target with its first *l* rows equal to *Y*_l_ and the rest equal to 0, and $C \in {\mathbb{R}}^{n \times n}$ is a (penalty) diagonal matrix with its first l diagonal elements $c_{ii} = C_{i}$ and the rest equal to 0.

Now let us solve the above optimization problem. We first compute the gradient of the objective function w.r.t. *W* and then by setting the gradient to zero, we obtain the optimal output weights (i.e., the SS-ELM solution) if *L* ≤ *l*:

8 $$W^{*} = ({\mathbf{I}}_{L} + H^{T} CH + \lambda H^{T} LH)^{ - 1} H^{T} C\tilde{Y}{\kern 1pt}$$

where I_*L*_ is an identity matrix of dimension *L*.

If *L* > 1 (common in SSL), the optimal output weights can be solved by the alterative form:

9 $$W^{*} = H^{T} ({\mathbf{I}}_{n} + CHH^{T} + \lambda LHH^{T} )^{ - 1} C\tilde{Y}{\kern 1pt} {\kern 1pt}$$

where **I**_n_ is an identity matrix of dimension *n*.

In summary, SS-ELM training algorithm consists of two key steps:

- Step 1: Initialize an ELM network of *L* hidden neurons with random input weights and biases, and calculate the output matrix of the hidden neurons $H \in {\mathbb{R}}^{n \times L}$.
- Step 2: Use Eq. (8) or (9) to compute the output weights *W*.

## Feature extraction algorithms

EEG feature extraction algorithms can be roughly divided into time-domain, frequency-domain, time–frequency, and nonlinear dynamics based analysis. This paper compares the discrete wavelet-packet transform (Alves et al. 2017) and Hilbert–Huang transform (Huang et al. 1996, 1998; Huang and Wu 2008, Huang and Shen 2014; Huang, Song, Gupta and Wu 2014; Krishna and Ramaswamy 2017) approaches for extracting the time–frequency features from the physiological signals.

### Wavelet-packet decomposition

Wavelet transform is a multi-scale signal analysis method. The method can characterize the local features of the signal in time and scale domain, so it is very suitable for the analysis of transient characteristics and time–frequency characteristics of non-stationary EEG signal.

Wavelet Packet Decomposition (WPD) is a generalization of wavelet decomposition. In the wavelet analysis, the approximation part is decomposed into the approximation part and detail part at another level. This process is repeated until the maximal number of decomposition levels is reached. However, in the WPD details are also decomposed. WPD has multi-scale characteristics and provides great choice for time–frequency analysis. In the multiresolution wavelet analysis, the Hilbert space $L^{2} ({\mathbb{R}})$ is decomposed into the sum of all orthogonal wavelet subspaces $W_{j} ({\text{scale factor }}j \in {\mathbb{Z}})$:

10 $$L^{2} ({\mathbb{R}}) = \mathop \oplus \limits_{{j \in {\mathbb{Z}}}} W_{j} {\kern 1pt}$$

**Fig. 2:**
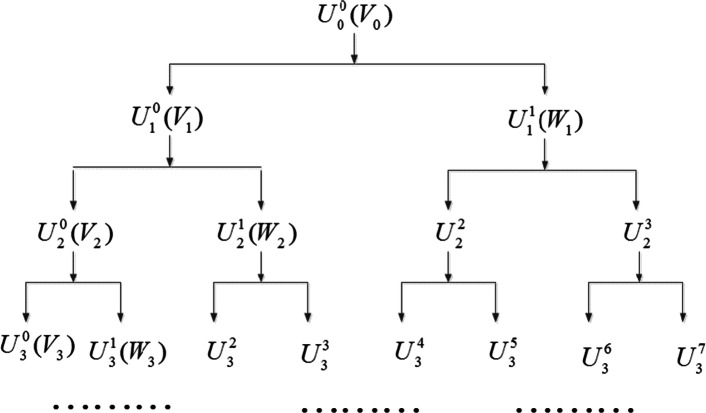
Illustration of the spatial WPD

WPD continues to dichotomize $W_{j} (j = 1,2, \ldots )$, as shown in Fig. 2, where $U_{j}^{n}$ is the wavelet-packet space of the scale *j* and its orthogonal basis $u_{j,k}^{n} (t) = 2^{ - j/2} u^{n} (2^{ - j} t - k)$(*k* is the translation factor) satisfies:

11 $$u_{j,0}^{n} (t) = \left\{ \begin{aligned} &\sum\limits_{k} {h_{0} (k)u_{j - 1,k}^{i} ,} \quad {\text{if }}n{\kern 1pt} {\kern 1pt} {\text{is even}} \hfill \\ & \sum\limits_{k} {h_{1} (k)u_{j - 1,k}^{j} } ,\quad {\text{ otherwise}} \hfill \\ \end{aligned} \right.$$

where $j,k \in {\mathbb{Z}},n = 0,1, \ldots ,2^{j} - 1,h_{0} (k){\text{ and }}h_{1} (k)$ are a pair of orthogonal mirror filters with the relationship $h_{1} (k) = ( - 1)^{1 - k} \cdot h_{0} (1 - k)$.

If *f*(*t*) is a function in the Hilbert space $L^{2} ({\mathbb{R}})$, when the scale is small enough we can approximate the coefficient $d_{0}^{0} (k)$ of the space $U_{0}^{0}$ by the sampling sequence $f(k\Delta t)$ or the normalized *f*(*k*). According to fast algorithm of WPD, the wavelet-packet coefficient of the *j*-th scale and *k*-th node can be expressed by:

12 $$d_{j}^{n} (k) = \left\{ \begin{aligned} &\sum\limits_{m} {h_{0} (m - 2k)d_{j - 1}^{n/2} (m)} , \quad {\text{if }}n{\kern 1pt} {\text{is even}} \hfill \\ &\sum\limits_{m} {h_{1} (m - 2k)d_{j - 1}^{(n - 1)/2} (m)} ,\quad {\text{ otherwise}} \hfill \\ \end{aligned} \right.{\kern 1pt} {\kern 1pt} {\kern 1pt}$$

In this way, we can get wavelet-packet coefficients of a signal at all scales. It is known that the EEG signals relevant to MWL are in the frequency band of [0–50] Hz. The 17-channel electrophysiological signals are decomposed into five levels. Using (12), we extract the spectral power of the first six nodes ([0–7.8], [7.8–15.6], [15.6–23.4], [23.4–31.2], [31.2–39], [39–46.8]) as the features of the EEG signal.

### Hilbert–Huang transform

The Hilbert–Huang transform (HHT) is a signal processing method suitable for nonlinear and nonstationary signal analysis and has been successfully applied to various fields, including geophysics and biomedicine (Huang et al. 1998, 2008, 2014). The core idea of the HHT is to use Empirical Mode Decomposition (EMD) to decompose a time-series signal into Intrinsic Mode Functions (IMFs) with a trend and then apply the Hilbert spectral analysis (HSA) method to the IMFs to obtain instantaneous frequency data. The HHT assumes that any signal is composed of a finite and often small number of components (described as IMFs), which form a complete and nearly orthogonal basis for the original signal.

#### Empirical mode decomposition

The general idea of the Empirical Mode Decomposition (EMD) method is to use the mean value of the upper and lower envelopes of the time series to determine the *instantaneous equilibrium*, and then extract the IMFs. The main steps of EMD include:

Step 1: Find the local maximum and minimum of the original sequence *x*(*t*), and connect the local maximum and the minimum with the cubic curve interpolation to obtain the maximum envelope $x_{\hbox{max} } (t)$ and the minimum envelope $x_{\hbox{min} } (t)$.

Step 2: Obtain the mean value *m*(*t*) by averaging $x_{\hbox{max} } (t)$ and $x_{\hbox{min} } (t)$ at each time instant.

Step 3: Calculate the difference between the original sequence *x*(*t*) and the instantaneous mean *m*(*t*), i.e.,

13 $$h(t) = x(t) - m(t){\kern 1pt}$$

For different datasets, *h*(*t*) may or may not be an IMF, which must satisfy the following conditions:

- C1: In the whole dataset, the number of extrema and the number of zero crossings must either be equal or differ at most by one.
- C2: At any point, the mean value of the two envelopes defined by the local maxima and local minima is zero.

If *h*(*t*) satisfies the above conditions, it is an IMF; otherwise *h*(*t*) is taken as the original sequence, and Steps 1–3 are repeated until C1 and C2 are satisfied.

The first IMF $c_{1} (t)$ should contain the finest scale or the shortest-period oscillation in the signal, which can be subtracted from the original sequence by:

14 $$x(t) - c_{1} (t) = r_{1} (t)$$

The residue $r_{1} (t)$ still contains longer-period variations. This residual signal is then treated as the new data and subjected to the same sifting process of the EMD to obtain an IMF of lower frequency. This procedure is repeatedly applied to all subsequent *r*_*j*_, yielding:

15 $$\begin{aligned} r_{1} (t) - c_{2} (t) = r_{2} (t), \\ r_{2} (t) - c_{3} (t) = r_{3} (t), \\ \vdots \\ r_{n - 1} (t) - c_{n} (t) = r_{n} (t) \\ \end{aligned}$$

The decomposition process finally stops when the residue $r_{n} (t)$ becomes a monotonic function or a function with only one extremum, from which no further IMF can be extracted. By summing up (14) and (15), we have:

16 $$x(t) = \sum\limits_{i = 1}^{n} {c_{i} (t)} + r_{n} (t){\kern 1pt} {\kern 1pt}$$

Therefore, after EMD the original signal is decomposed into *n* IMFs $(c_{1} (t),c_{2} (t), \ldots ,c_{n} (t))$ and a residue $r_{n} (t)$, which can be either the adaptive trend or a constant.

#### Hilbert spectral analysis

The Hilbert transform of any function *x*(*t*) of *L*^*P*^ class is defined as the convolution of *x*(*t*) with function $h(t) = \frac{1}{\pi t}$:

17 $$y\left( t \right) = H[x(t)] = \frac{1}{\pi }P\int\limits_{ - \infty }^{\infty } {\frac{x(\tau )}{t - \tau }} d\tau$$

where *P* is the Cauchy principal value of the singular integral.

With the Hilbert transform *y*(*t*) of the function *x*(*t*), we obtain the analytic function,

18 $$z(t) = x(t) + jy\left( t \right) = A(t)e^{j\theta (t)}$$

where $j = \sqrt { - 1} ,A(t) = \sqrt {x^{2} (t) + y^{2} \left( t \right)} {\kern 1pt}$ is the instantaneous amplitude, and $\theta (t) = \arctan \left[ {\frac{y(t)}{x(t)}} \right]$ is the instantaneous phase function.

An IMF after the Hilbert transform can be expressed in Eq. (18). If we perform a Fourier transform on *z*(*t*), we have:

19 $$W\left( \omega \right) = \int_{ - \infty }^{\infty } {A\left( t \right)} e^{j\theta \left( t \right)} e^{ - j\omega t} dt = \int_{ - \infty }^{\infty } {A\left( t \right)} e^{{j\left( {\theta \left( t \right) - \omega t} \right)}} dt$$

Then by the stationary phase method (Copson 1967), the maximum contribution to *W*(*ω*) is given by the frequency satisfying the condition:

20 $$\frac{d}{dt}\left( {\theta \left( t \right) - \omega t} \right) = 0$$

Therefore the best instantaneous frequency is simply $\omega (t) = \frac{d\theta (t)}{dt}$.

**Fig. 3:**
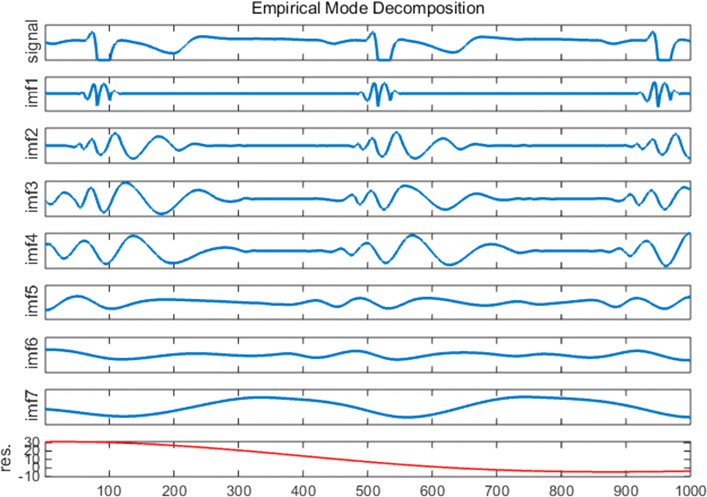
The EMD of a 2 s ECG segment into seven IMFs (subject A)

Figure 3 illustrates the decomposition of a 2 s ECG data into seven IMFs and a residue (trend term).

With both amplitude and frequency being a function of time, we can express the amplitude (or energy, the square of amplitude) in terms of a function of time and frequency, $H\left( {\omega ,t} \right)$. The marginal spectrum can then be defined as:

21 $$h\left( \omega \right) = \int_{0}^{T} {H\left( {\omega ,t} \right)dt}$$

where [0, *T*] is the temporal domain over which the data is defined. The marginal spectrum represents the accumulated amplitude (energy) over the entire data span in a probabilistic sense and provides a measure of the total amplitude (energy) contribution from each frequency value, serving as an alternative spectrum expression of the data to the traditional Fourier spectrum.

As pointed out by Huang et al. (1996, 1998), the frequency in either $H\left( {\omega ,t} \right)$ or *H*(*ω*) has a totally different meaning from the Fourier analysis. In the Fourier representation, the existence of energy at a frequency *ω* means a component of sine or cosine wave persisted through the time course of the data. Here, the existence of energy at the frequency *ω* means only that, in the whole time course of the data, there is a higher likelihood for such a wave to have appeared locally. In fact, the Hilbert spectrum is a weighted non-normalized joint amplitude-frequency-time distribution. The weight assigned to each time–frequency cell is the local amplitude. Consequently, the frequency in the marginal spectrum indicates only the likelihood that an oscillation with such a frequency exists. The exact occurrence time of that oscillation is given in the full Hilbert spectrum.

In addition to the marginal spectrum, we can also define the Instantaneous Energy (IE) density level as:

22 $$IE\left( t \right) = \int_{\omega } {H^{2} \left( {\omega ,t} \right)d\omega }$$

Obviously, IE also depends on time; it can be used to check the energy fluctuation.

In summary, the signal *x*(*t*) is decomposed by EMD method into $c_{i} (t){\kern 1pt} {\kern 1pt} {\kern 1pt} {\kern 1pt} {\kern 1pt} (i = 1,2, \ldots ,n)$, which can be expressed as:

23 $$c_{i} (t) = {\text{Re}}[A_{i} (t)\exp (j\int {\omega_{i} (t)dt} )]{\kern 1pt} {\kern 1pt}$$

where Re[·] represents the real part of terms with brackets and $A_{i} (t)$ is represented on the time–frequency plane.

We obtain the Hilbert spectrum of $c_{i} (t)$ as:

24 $$H_{i} (\omega ,t) = \left\{ {\begin{array}{*{20}l} {A_{i} (t){\kern 1pt} {\kern 1pt} {\kern 1pt} ,{\kern 1pt} {\kern 1pt} {\kern 1pt} {\kern 1pt} {\text{if }}\omega = \omega_{i} (t)} \hfill \\ {0{\kern 1pt} {\kern 1pt} {\kern 1pt} ,{\kern 1pt} {\kern 1pt} {\kern 1pt} {\kern 1pt} {\kern 1pt} {\kern 1pt} {\kern 1pt} {\kern 1pt} {\kern 1pt} {\kern 1pt} {\kern 1pt} {\kern 1pt} {\kern 1pt} {\kern 1pt} {\kern 1pt} {\kern 1pt} {\kern 1pt} {\kern 1pt} {\text{otherwise}}} \hfill \\ \end{array} } \right.{\kern 1pt} {\kern 1pt}$$

The IMFs obtained by sifting process of EMD constitute an adaptive basis. This basis usually satisfies empirically all the major mathematical requirements for a time series decomposition method, such as convergence, completeness, orthogonality, and uniqueness, as discussed by Huang et al. (1998).

For an arbitrary time series of length *N*, *x*(*t*), if EMD is used to obtain its IMF components and instantaneous frequencies and instantaneous amplitudes of those IMFs are obtained by using the Hilbert transform, *x*(*t*) can be expressed as:

25 $$x(t) = \text{Re} [\sum\limits_{i = 1}^{n} {A_{i} (t)\exp (j\int {\omega_{i} (t)dt} )} ]{\kern 1pt} {\kern 1pt}$$

Here the residue, *r*_*n*_, is not expressed in terms of a simple oscillatory form for it is either a monotonic function or a function with only one extrema not containing enough information to check if it is an oscillatory component is physically meaningful.

Equation (24) gives both amplitude and frequency of each component as functions of time. The Fourier representation of the same data is

26 $$x\left( t \right) = \text{Re} \sum\limits_{i = 1}^{\infty } {A_{i} e^{{j\omega_{i} t}} }$$

where both *A*_*i*_ and *ω*_*i*_ are constants.

The difference between Eqs. (25) and (26) is fundamental: the IMF represents largely a generalized Fourier expansion. The variable amplitude and instantaneous frequency not only improve the efficiency of the expansion, but enable the expansion to accommodate nonlinear and nonstationary variations in data. The IMF expansion lifts the restriction of constant amplitude and fixed frequency in the Fourier expansion, allowing for a variable amplitude and frequency representation over time. Equation (25) also enables us to represent the amplitude and the instantaneous frequency as functions of time in a 3D plot, in which the amplitude can be contoured on the frequency-time plane. This frequency-time distribution of the amplitude is designated as the Hilbert amplitude spectrum, $H(\omega ,t)$, or simply Hilbert spectrum. If amplitude squared is more desirable commonly to represent energy density, then the squared values of amplitude can be substituted to produce the Hilbert energy spectrum as well.

The Hilbert amplitude spectrum $H(\omega ,t)$ of the original signal *x*(*t*) can be expressed as:

27 $$H(\omega ,t) = \sum\limits_{i = 1}^{n} {H_{i} (\omega ,t)} {\kern 1pt} {\kern 1pt}$$

The calculation of sample entropy requires smaller time span of either deterministic or random signal and is thus more computationally efficient. In addition, it is insensitive to the noises. Thus in addition to the HHT-derived features, we also include sample entropy as an additional feature. Specifically, we calculate the variance contribution of each IMF component as well as its correlation coefficient with the original signal, select the first three IMF components with the highest contribution, and then compute their respective sample entropies, which are used jointly with the energy spectrum entropy and the Hilbert marginal spectral entropy as the 5-dim. feature vector of a sample data.

## Data acquisition and data preprocessing

### Subjects

Six subjects (22–24 y/o, male; coded by A, B, C, D, E, and F) participated in the experiments. All subjects were healthy, had normal vision and dextromanual. Before the experiments, all subjects were informed of goals and procedure of the experiment and were trained for more than 10 h on aCAMS-based task operations.

### Experimental tasks

The simulated task platform used in our experiments is automated-enhanced Cabin Air Management System (aCAMS), which consists of four subsystems: concentration of oxygen (O_2_), air pressure (P), concentration of carbon dioxide (CO_2_), and temperature (T). In the experiment, we used the aCAMS to simulate the task environment in a closed cabin. The operator’s MWL is mainly affected by the Number Of Subsystems (NOS) assigned to him for manual control and the Actuator Sensitivity (AS) in the manual control systems. The aCAMS simulation platform constitutes a complex human–machine cooperative task environment. Nihon Kohden^®^ measurement system was used to measure physiological signals at a sampling rate of 500 Hz.

### Experimental procedure

The aCAMS system has four subsystems, each having two control modes: automatic or manual control. The two modes of control can be switched arbitrarily. The control objective of the experiment is to maintain the output variables of the four subsystems within their target ranges by automatic control by automation systems, manual control by human operator, or a mixture of both modes. For manual control, there are two levels of actuator sensitivity (AS): Low or High. The sensitivity of the control variable under High AS is higher than Low AS (Wang et al. 2016).

**Fig. 4:**
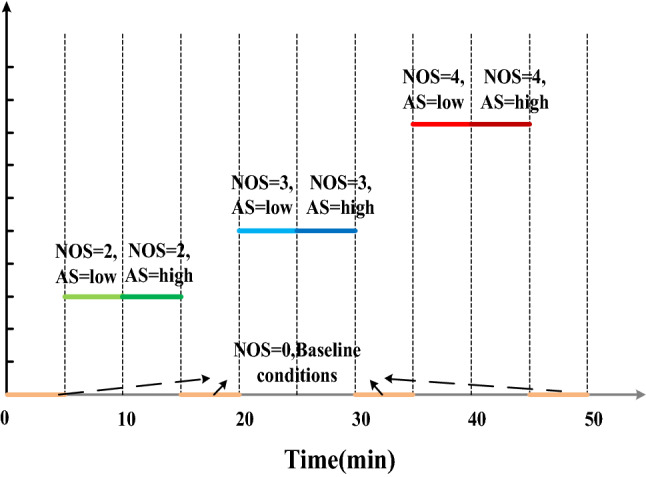
The 10 task-load conditions involved in each session of experiment

Each session lasts for 50 min. and consists of 10 different task-load conditions. The conditions #1, 4, 7, and 10 are under automatic control mode. Operator manually controls two subsystems (O2 and P) in the conditions #2 and 3, the only difference between the two conditions is that the AS is different. Figure 4 illustrates the 10 task-load conditions in a session of experiment. During the last 10 s of each condition, the operator performs self-assessment of his performance in that condition, so we only consider the measured data of 290 s per condition.

**Fig. 5:**
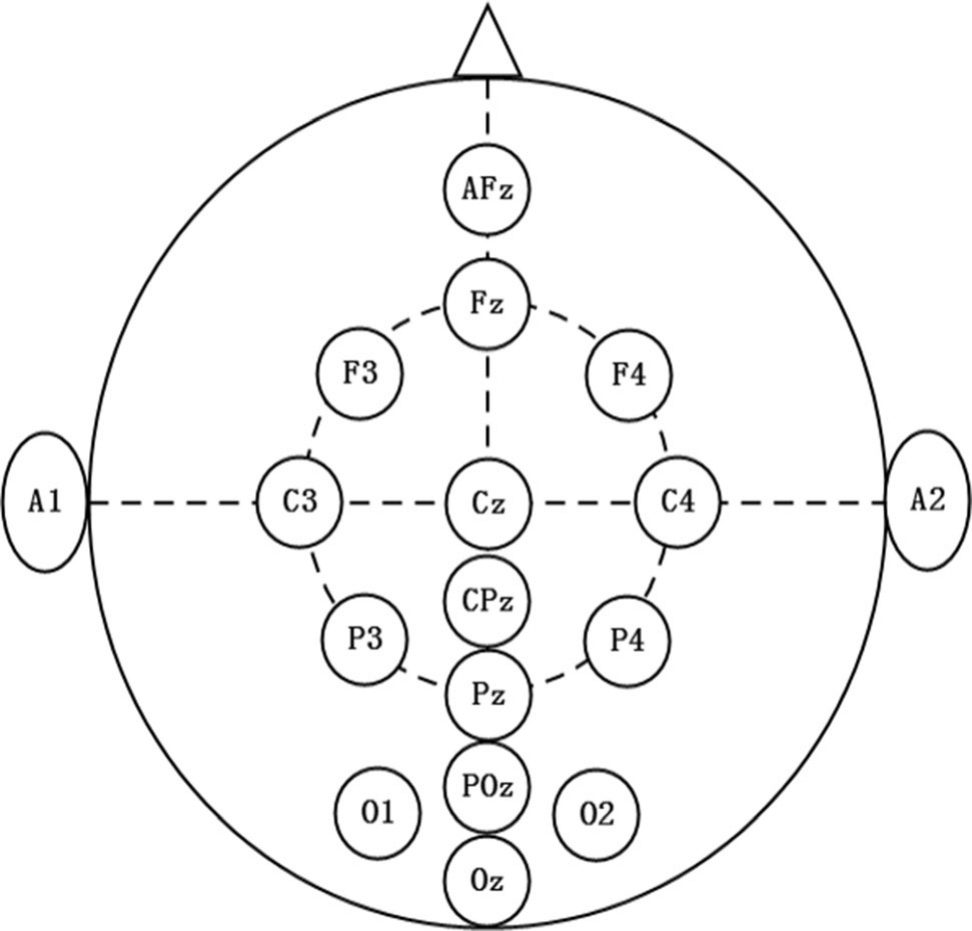
The 15 EEG measurement electrodes used in our experiments

The EEG, ECG and EOG signals for each subject were collected during the aCAMs operation by using a signal acquisition instrument (sampling rate: 500 Hz). The instrument has the function of removing the disturbance of the power–frequency on the electrophysiological signals. In the international standard 10–20 EEG electrode placement system (Okamoto et al. 2004), 15 electrodes that are most relevant to the MWL variations were selected, namely F3, F4, C3, C4, P3, P4, O1, O2, Fz, Cz, CPz, Pz, AFz, POz, and Oz (Yin and Zhang 2014a, b). The placement of the EEG measurement electrodes is shown in Fig. 5, in which the earlobes A1 and A2 were used as the referential potential. In addition, the potential difference between the upper middle part of the clavicle and the lower middle part of the left rib was recorded as ECG signal. The EOG signal was measured by the potential between the electrodes above and below the left eye. The recorded raw signals is filtered by a Butterworth band-pass filter (0–40 Hz) and the coherent method is used to remove the eye artifacts.

### Physiological data labeling

**Fig. 6:**
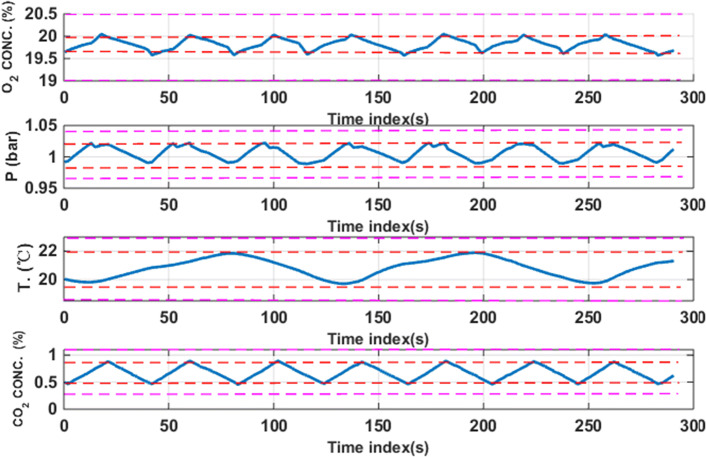
The outputs of four subsystems under human-computer shared control (subject A)

The preprocessed data is divided by the sliding time window with length of 1 s (with no overlapping), then each load condition contains 290 sample data. In addition to physiological data, the experiment also records the task performance data, i.e., the output of the subsystems under control. Performance data for subject A is shown in Fig. 6.

**Fig. 7:**
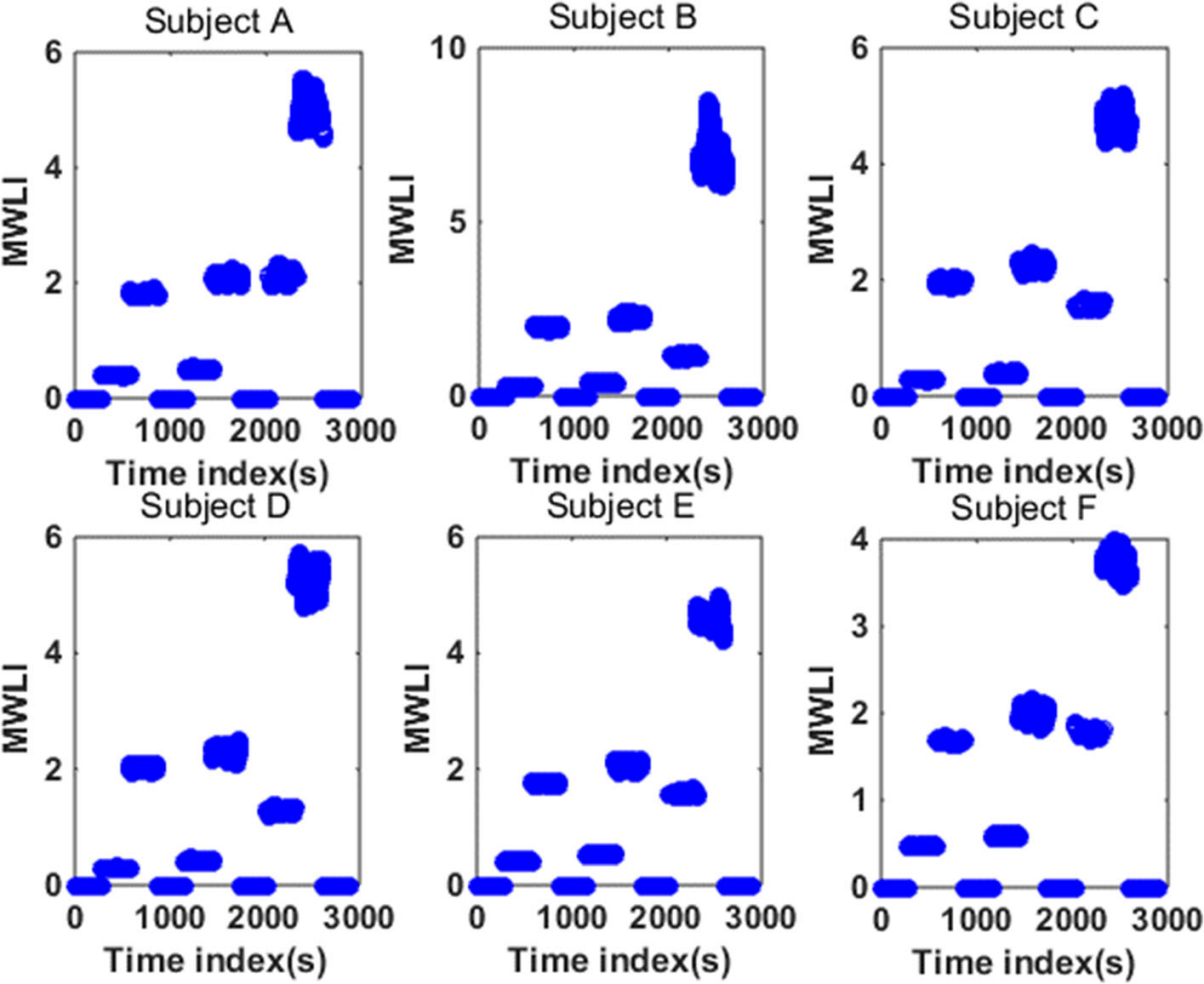
The temporal variation of MWLI for each subject

Where the area between the red lines is the target range and the area between the pink lines is the safe range. In order to quantify the MWL level, we define the Mental Workload Index (MWLI):

28 $$MWLI = w_{{O_{2} }} r_{{O_{2} }} (t) + w_{P} r_{P} (t) + w_{{CO_{2} }} r_{{CO_{2} }} (t) + w_{T} r_{T} (t)$$

where *r*(*t*) with different subscript is Boolean variable of the corresponding subsystem (when the output of the corresponding subsystem is in target range at time *t*, $r(t) = 0$; otherwise $r(t) = 1$) and *w* represents the weight of the corresponding subsystem that can be determined by:

29 $$w = w_{1} w_{2} w_{3}$$

where *w*_1_ represents the control weight of the corresponding subsystem (when the subsystem is under manual control, *w*_1_ = 1; otherwise *w*_1_ = 0), *w*_2_ represents the difficulty level of the corresponding subsystem among four subsystems, and *w*_3_ denotes the difficulty level of control of the corresponding subsystem with different level of AS. More specifically, *w*_2_ and *w*_3_ are determined using the entropy method, as shown in Tables 1 and 2. The basic idea of entropy method is to determine the weight according to the indicator variability. In general, the smaller the information entropy of an indicator, the greater the variation in the indicator, the greater the amount of information provided, the greater the weight. By using (11) and (12), we obtain the second-to-second MWLI variations, as shown in Fig. 7. We can see that there exists individual difference across 6 subjects, but the overall trend of change is similar, for example, condition #9 has the peak (highest) level of MWL. The MWL level is higher in the conditions #3, 6 and 8, while the MWL level in the condition #2 and 5 is lower. The baseline conditions #1, 4, 7, and 10 are under automatic control, thus the MWL level in those 4 baseline conditions is zero (under-loaded). Based on those observations, we will classify the MWL into three classes (baseline, low, high) or four classes (baseline, low, medium, high).

**Table 1:** The weights assigned to the four subsystems for each subject

Subject	O_2_	P	CO_2_	T
A	0.1053	0.1041	0.5819	0.2087
B	0.1073	0.1110	0.5258	0.2559
C	0.1066	0.1100	0.6061	0.1773
D	0.1102	0.1170	0.5910	0.1818
E	0.1034	0.1072	0.6167	0.1727
F	0.1031	0.1050	0.6495	0.1424

**Table 2:** The different weights assigned to the four subsystems under binary (low vs. high) levels of Actuator Sensitivity (AS)

Subject	AS	O_2_	P	CO_2_	T
A	Low	0.1644	0.3824	0.2716	0.3656
	High	0.8356	0.6176	0.7284	0.6344
B	Low	0.1117	0.1408	0.2933	0.1416
	High	0.8883	0.8592	0.7067	0.8584
C	Low	0.1178	0.1724	0.2736	0.3146
	High	0.8822	0.8276	0.7264	0.6854
D	Low	0.1263	0.1740	0.2886	0.2217
	High	0.8737	0.8260	0.7114	0.7783
E	Low	0.1859	0.2908	0.2447	0.2886
	High	0.8141	0.7092	0.7553	0.7114
F	Low	0.2130	0.3431	0.2401	0.4038
	High	0.7870	0.6569	0.7599	0.5962

## Classification results and discussion

In this section, we present the SSL performance across the six subjects. We use the WPD and HHT algorithms to extract the relevant features. The effectiveness of SSL algorithm for MWL classification problem is validated by the pertinent empirical results. In addition, we examine the effect of the number of training data and the number of unlabeled data on the performance of SSL algorithm. Finally, we compare the performance of SSL algorithm and the commonly used supervised learning algorithms for OFS analysis.

In this study we utilized a windowing approach with a sliding time window with length of 1 s. The methods described in “Semi-supervised learning method” section were applied to the windowed data to extract features. The Daubechies wavelet (db4) function was used to decompose the EEG signals by using 5-level WPD. In this way, we can obtain a dataset of 2900 feature data with a feature dimensionality of 102 (= 6 features/channel × 17 channels). For the HHT algorithm, the sample entropies of the first three IMF components with the highest contribution to the variance, energy spectrum entropy and the Hilbert marginal spectral entropy were taken as the statistics-based features, hence we can get a dataset of 2900 samples with a feature dimensionality of 85 (= 5 features/channel × 17 channels).

Usually there are significantly more unlabeled data than the labeled data, thus we divide the dataset into labeled data and unlabeled data at the rate of 1:9. Labeled data is further divided into training and test data at the rate of 4:1. As a result, the number of training samples, test samples and unlabeled samples are 232, 58, and 2610, respectively.

To avoid the impact of the smaller test set on the ability of the SSL algorithm to effectively utilize labeled and unlabeled data (i.e., unbalanced data classification problem), we divide the dataset into labeled data and unlabeled data at the rate of 1:4. The labeled data is equally divided into training and testing data. Finally, the number of training samples, test samples and unlabeled samples are 290, 290, and 2320, respectively.

### Classification results

**Fig. 8:**
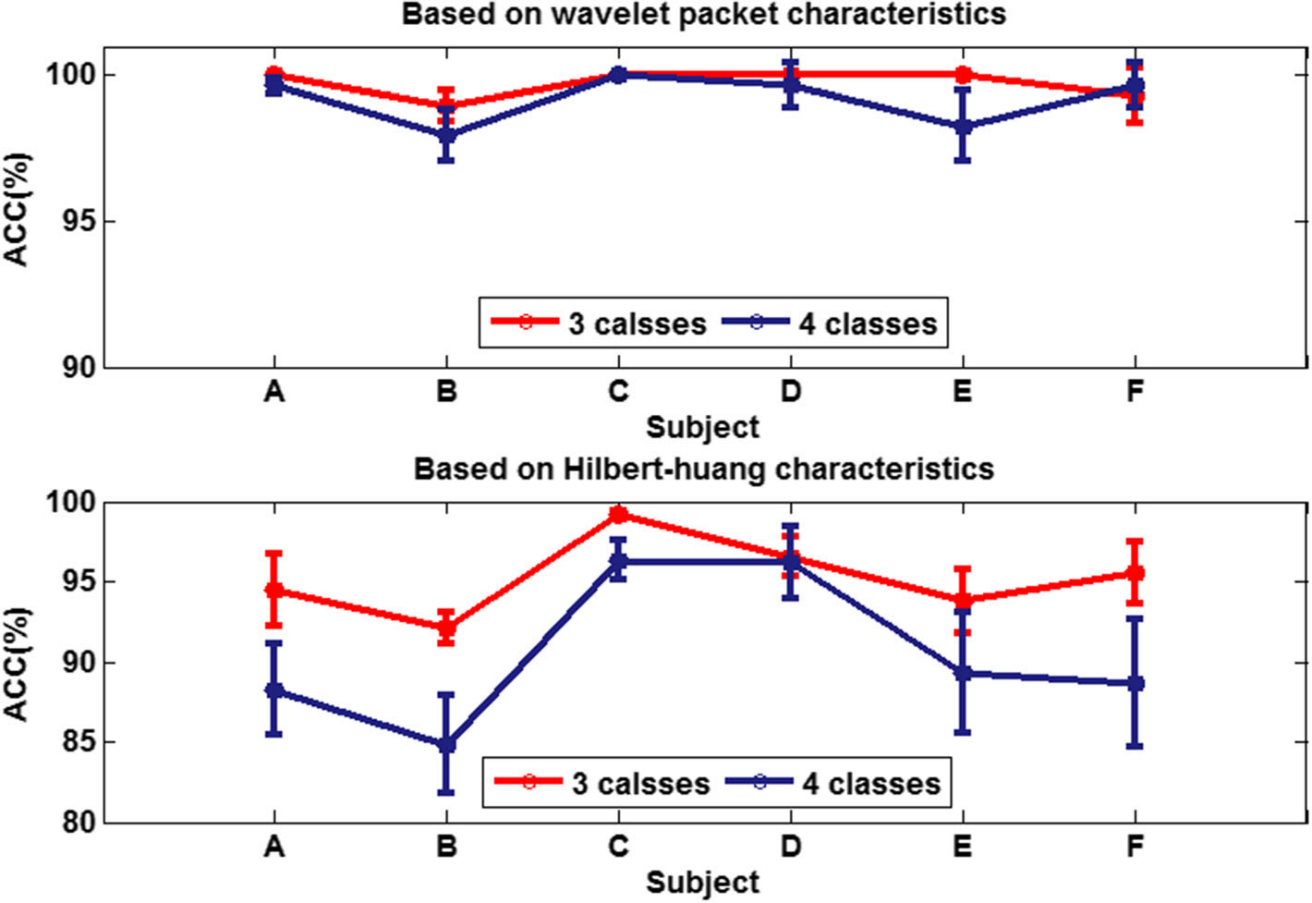
The testing classification accuracy

In this section, the SS-ELM is applied to classify MWL. As shown in Fig. 8, the proposed algorithm has satisfactory classification performance on the MWL-related dataset. In addition, the testing classification accuracy of both feature extraction algorithms exceeds 85%. In comparison, the WPD algorithm outperforms HHT algorithm, achieving a 3-class classification accuracy of 99.71% and 4-class accuracy of 99.19%.

In terms of individual differences (i.e., cross-subject variability), for both feature extraction algorithms, the worst classification performance is obtained for subject B among the six subjects, whereas the classification performance for subject C is the most stable in both three- and four-class cases.

**Fig. 9:**
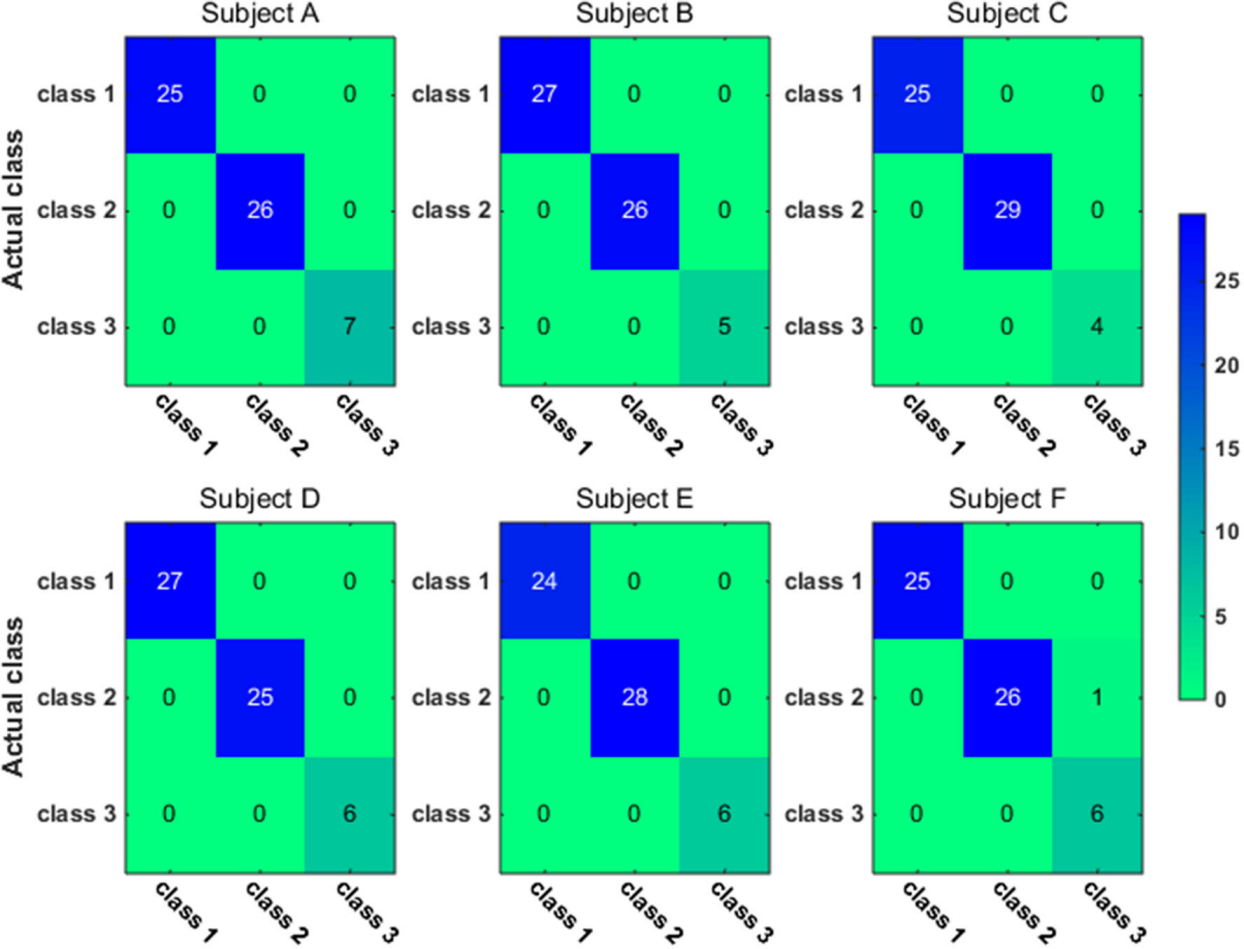
The testing classificaion confusion matrix using wavelet packet features (3-class case)

**Fig. 10:**
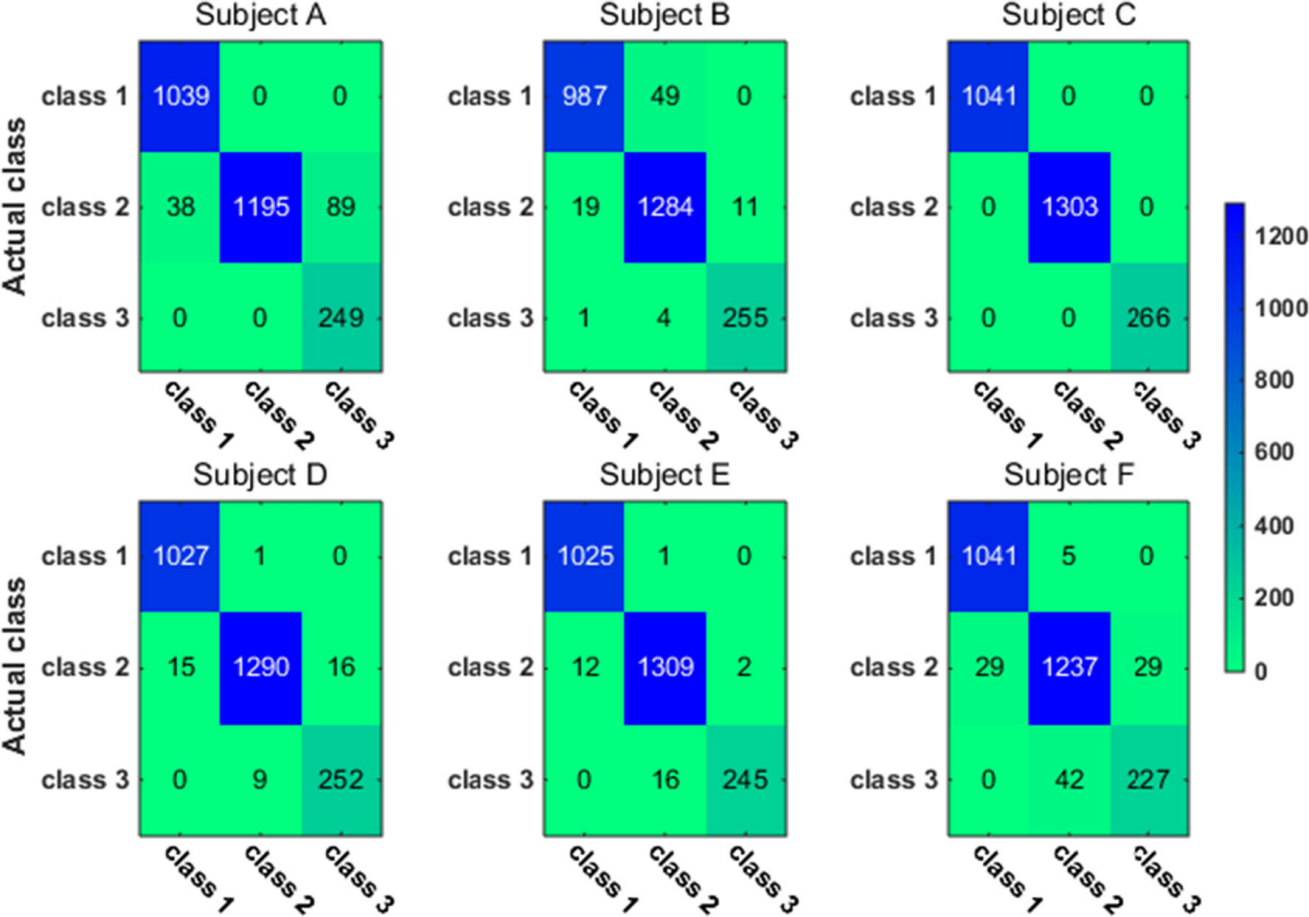
The classification confusion matrix calculated on unlabeled dataset using wavelet packet features (3-class case)

**Fig. 11:**
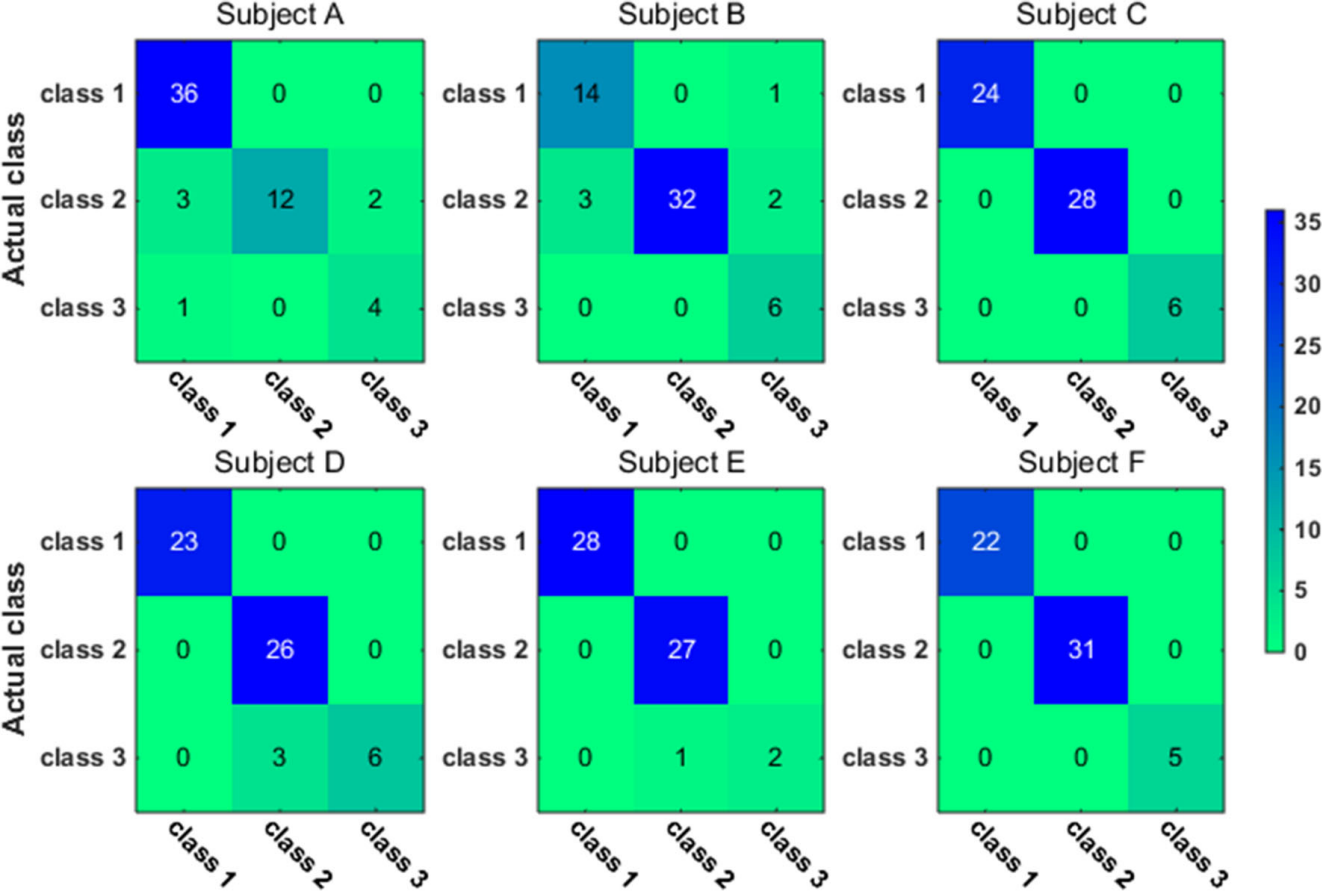
The testing classification confusion matrix using HHT features (3-class case)

**Fig. 12:**
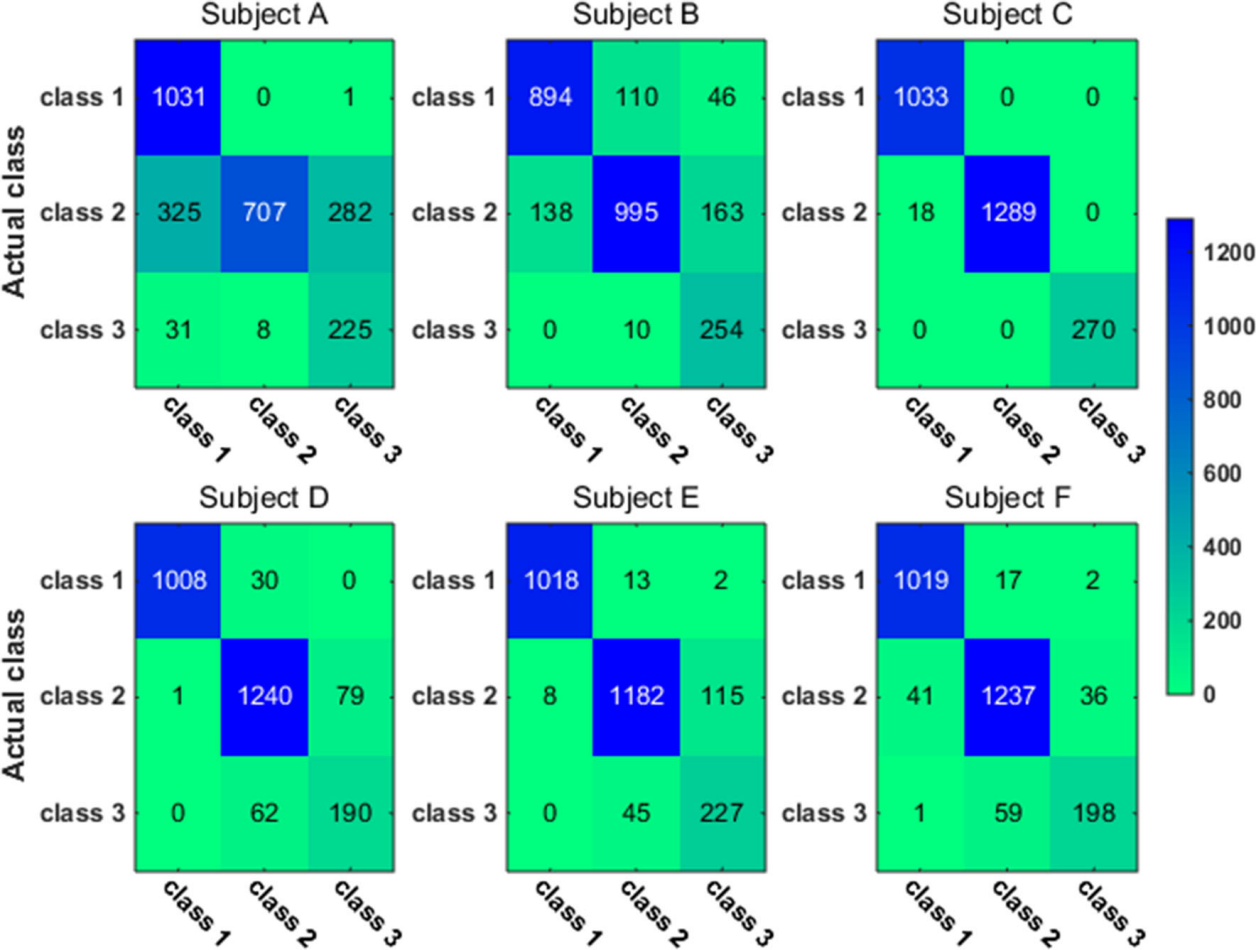
The classification confusion matrix calculated on unlabeled dataset using HHT features (3-class case)

**Fig. 13:**
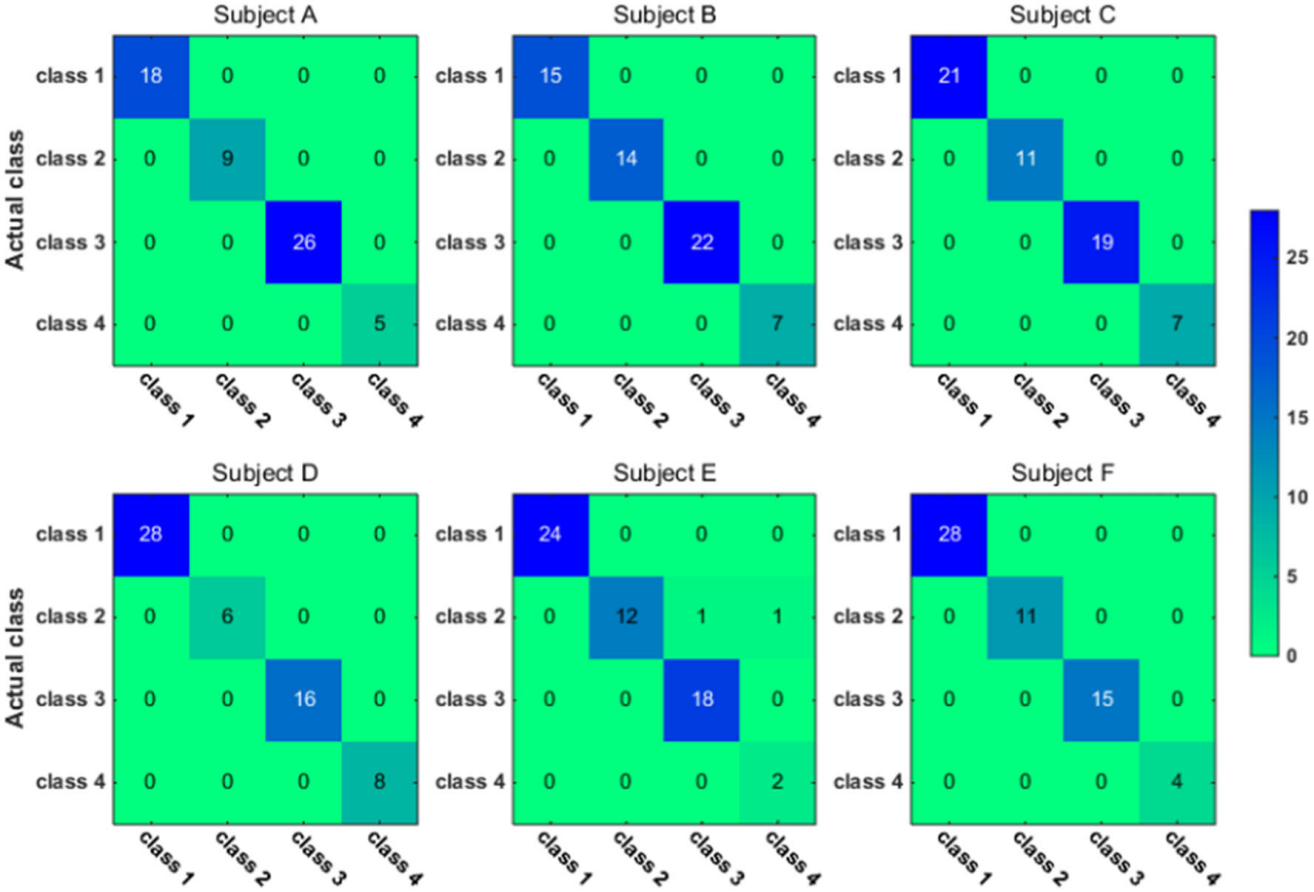
The testing classification confusion matrix using wavelet packet features (4-class case)

**Fig. 14:**
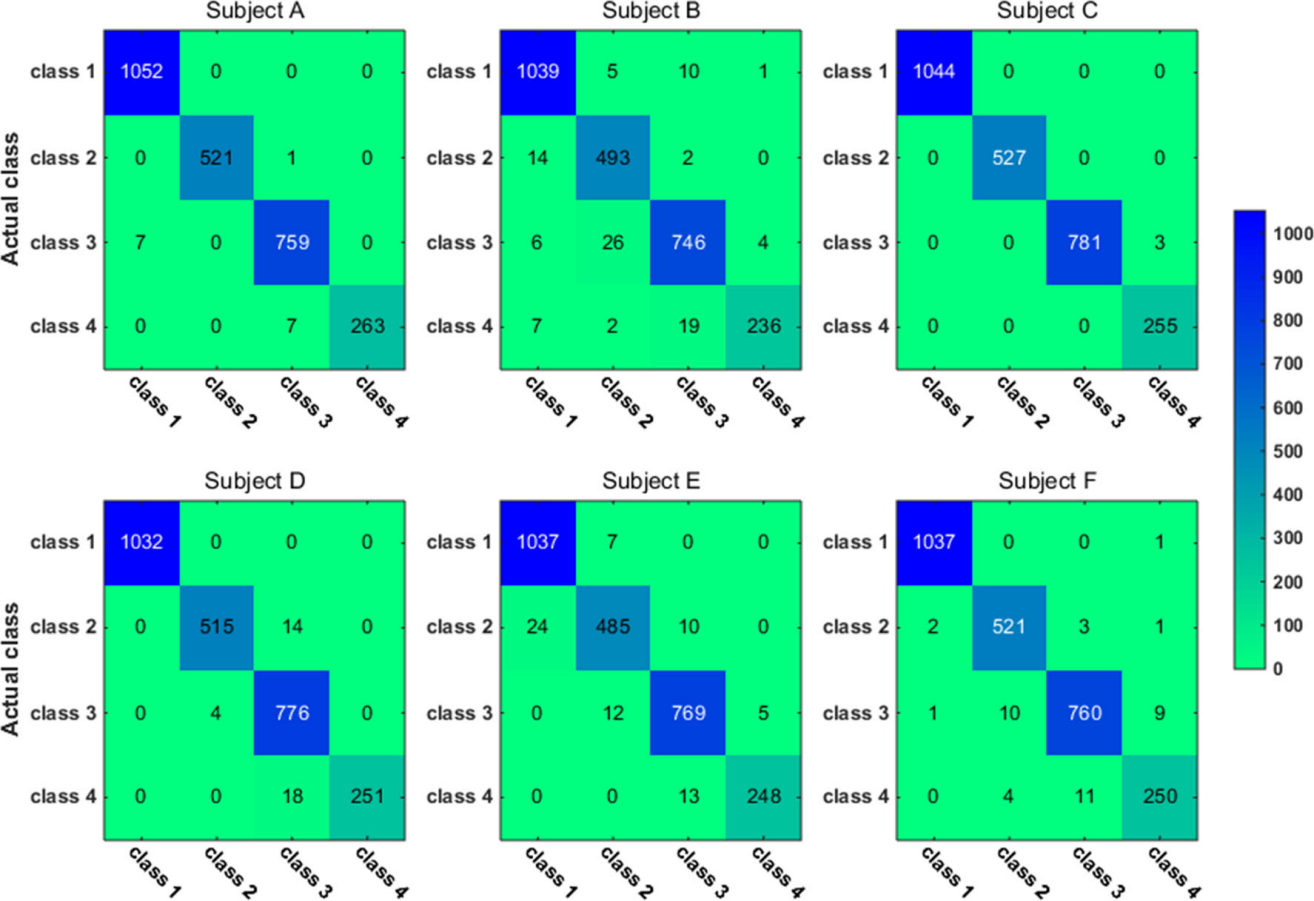
The classification confusion matrix calculated on unlabeled dataset using wavelet packet features (4-class case)

**Fig. 15:**
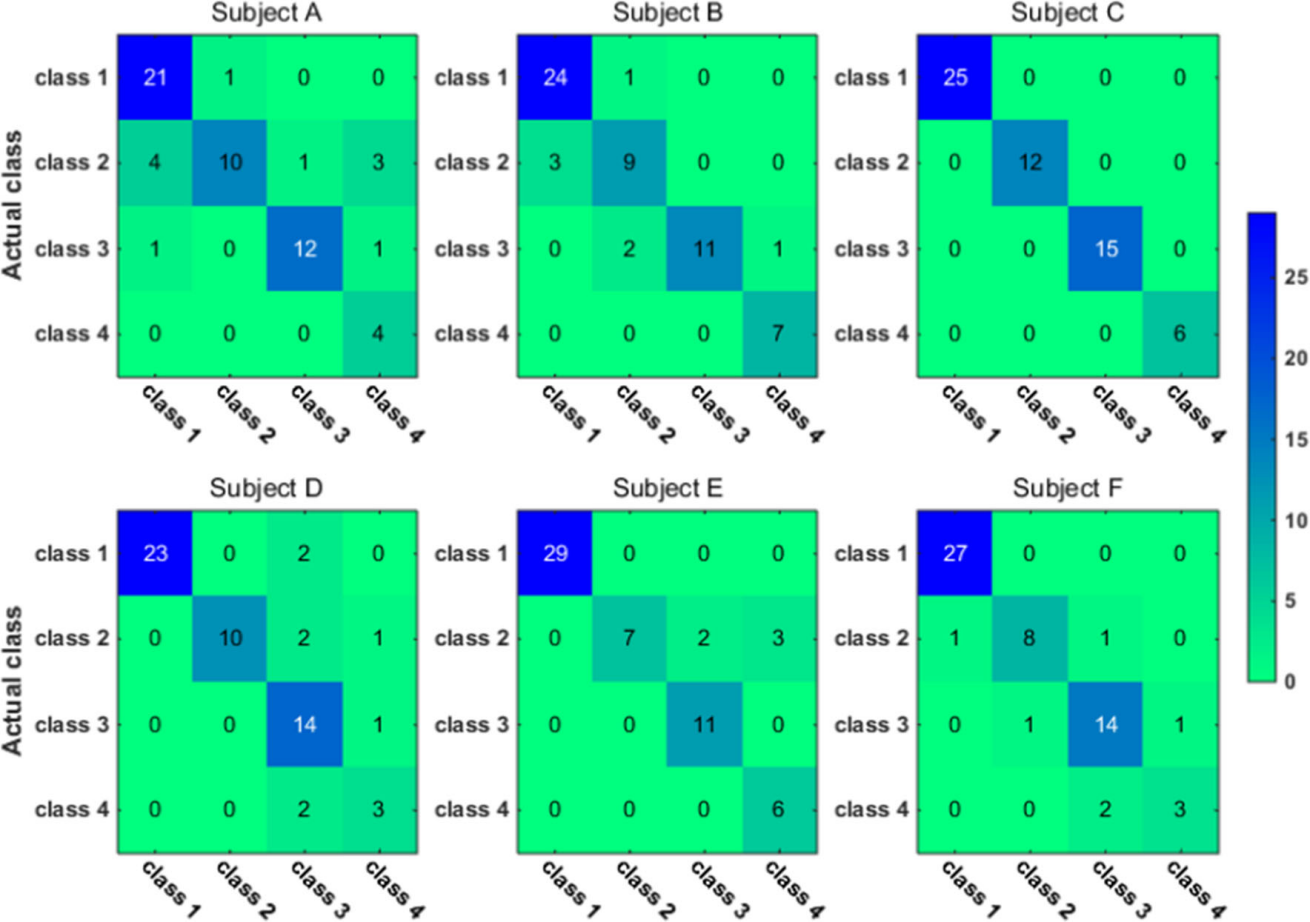
The testing classification confusion matrix using HHT features (4-class case)

**Fig. 16:**
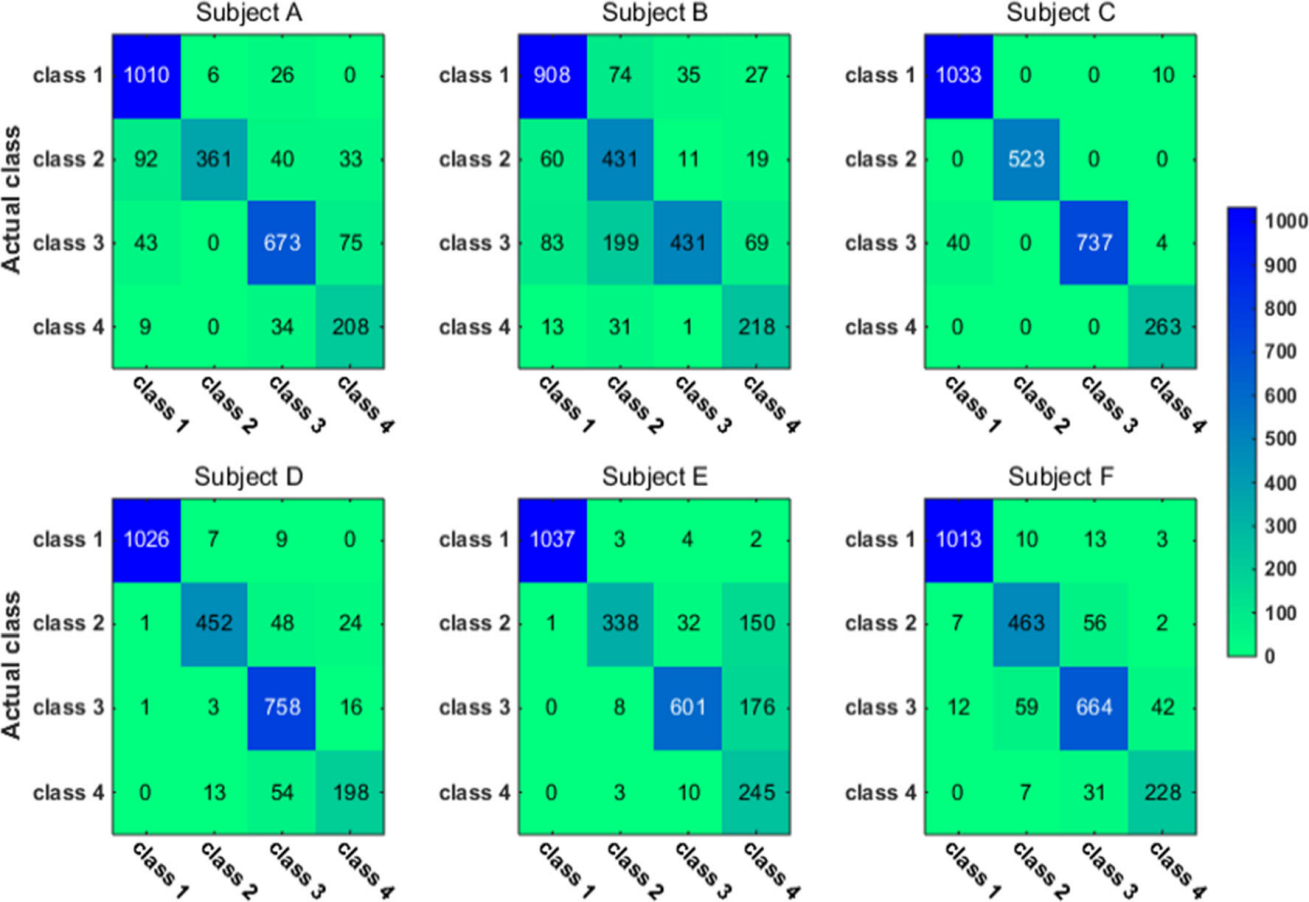
The classification confusion matrix calculated on unlabeled dataset using HHT features (4-class case)

To have a closer look at the classification accuracy for individual classes, we also present the classification confusion matrix for each subject in Figs. 9, 10, 11 and 12. Figures 9 and 10 give the confusion matrix resulted from the use of wavelet-packet features and computed on test set and unlabeled set respectively, while Figs. 11 and 12 show the confusion matrix resulted from the use of HHT-derived features and computed on test set and unlabeled set respectively. Additionally, we give the 4-class confusion matrix for each subject in Figs. 13, 14, 15 and 16. We can see that the confusion matrix result is comparable to (only slightly lower than) that for the 3-class problem.

Overall, using either wavelet-packet- or HHT-derived features, SSL algorithm leads to promising classification performance. From the confusion matrix, we can find that for the 3-class problem, it is relatively easier to classify Class1 and Class2 than Class3. Moreover, there is marked individual difference between subject B and C.

### Discussions

#### Labeled Sample Size

From the results shown in Fig. 8, we find that the WPD is more accurate and efficient for MWL classification task. Therefore, subsequently we focus on the discussion of the performance of the combination of wavelet-packet feature extraction and SS-ELM classification algorithms.

**Fig. 17:**
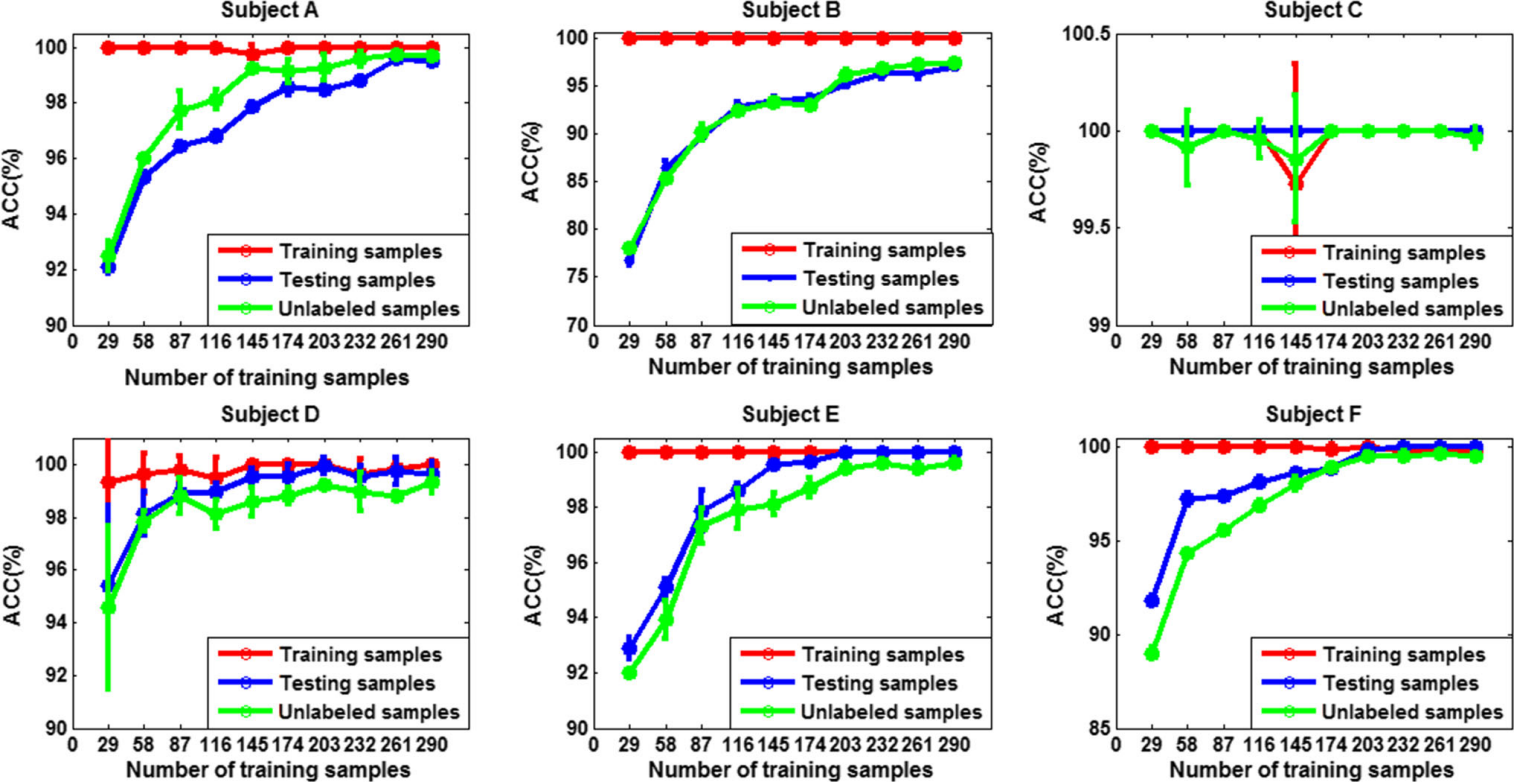
The classification accuracy versus size of training set for each subject. The error bars indicate standard deviation (3-class case)

**Fig. 18:**
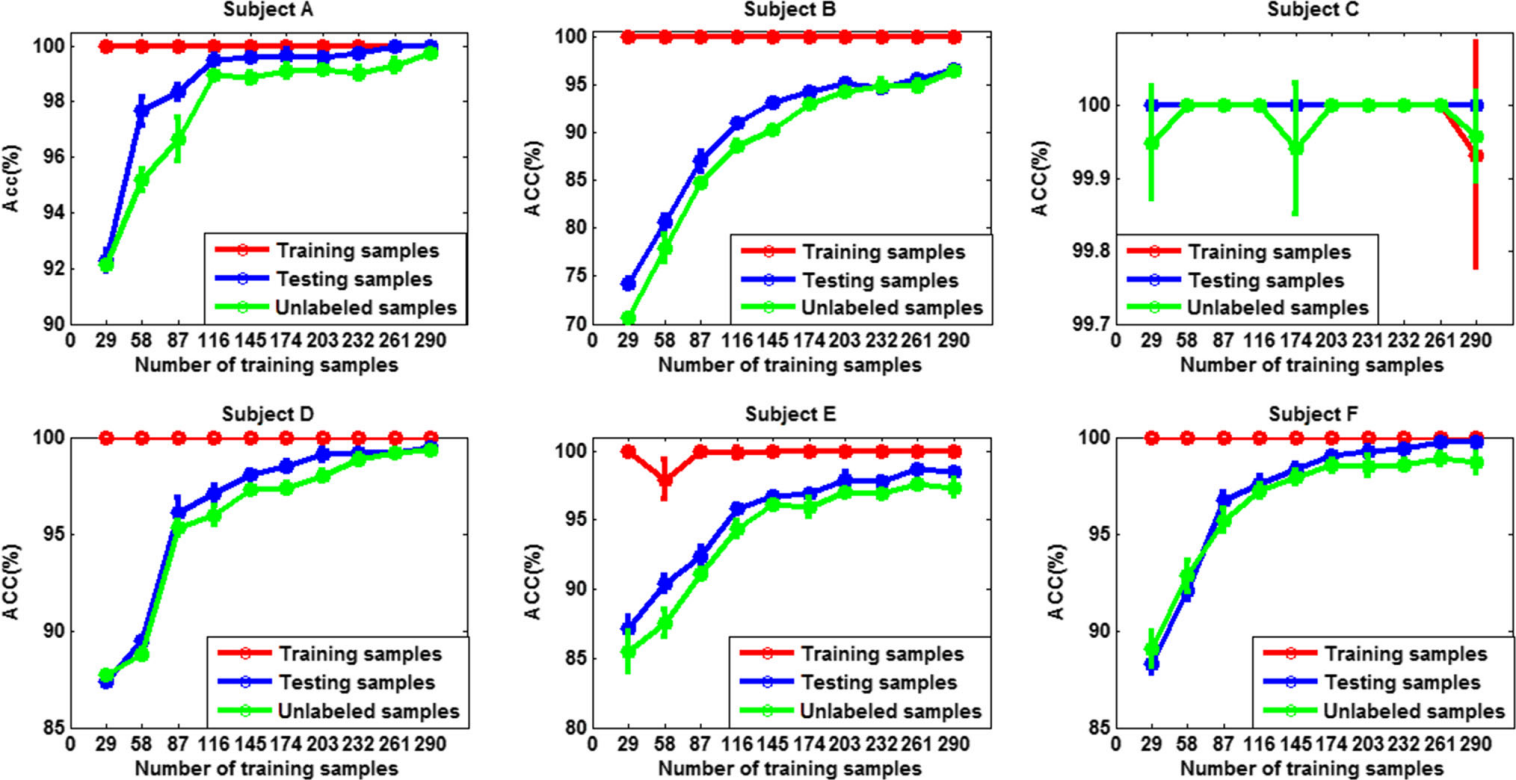
The classification accuracy versus size of training set for each subject (4-class case)

To test the effect of the number of labeled training data on the performance of SS-ELM algorithm, we gradually increased the size of training set, while fixing the size of both the unlabeled and testing set to 2630. The training and test accuracy as well as the accuracy calculated on unlabeled data for each subject are shown in Figs. 17 and 18 for 3- and 4-class problem, respectively [mean ± standard deviation (sd)].

We can see that except for subject C, the classification performance for other five subjects improves with the increase of the number of labeled data. For subject C, when the size of training set is 29, the accuracy already approaches 100%, so with the increase of the number of training samples, there is little room for further improvement of the accuracy. Therefore, we may conclude that satisfactory classification results can be obtained by using only a small number of labeled data. For other subjects, if training samples are scarce/sparse, the increase of the number of training samples has a great impact on the accuracy of the algorithm; However, if the training set is larger, the accuracy of the algorithm would improve little or stops improving with continued increase of the number of training samples. In summary, the benefit of SSL algorithm is reflected the best in the situations where only little labeled data is available.

#### Size of unlabeled dataset

To test the capacity of the graph-based SSL algorithm in utilizing unlabeled data, we gradually increase the number of unlabeled data, while fixing the size of labeled set to 29.

**Fig. 19:**
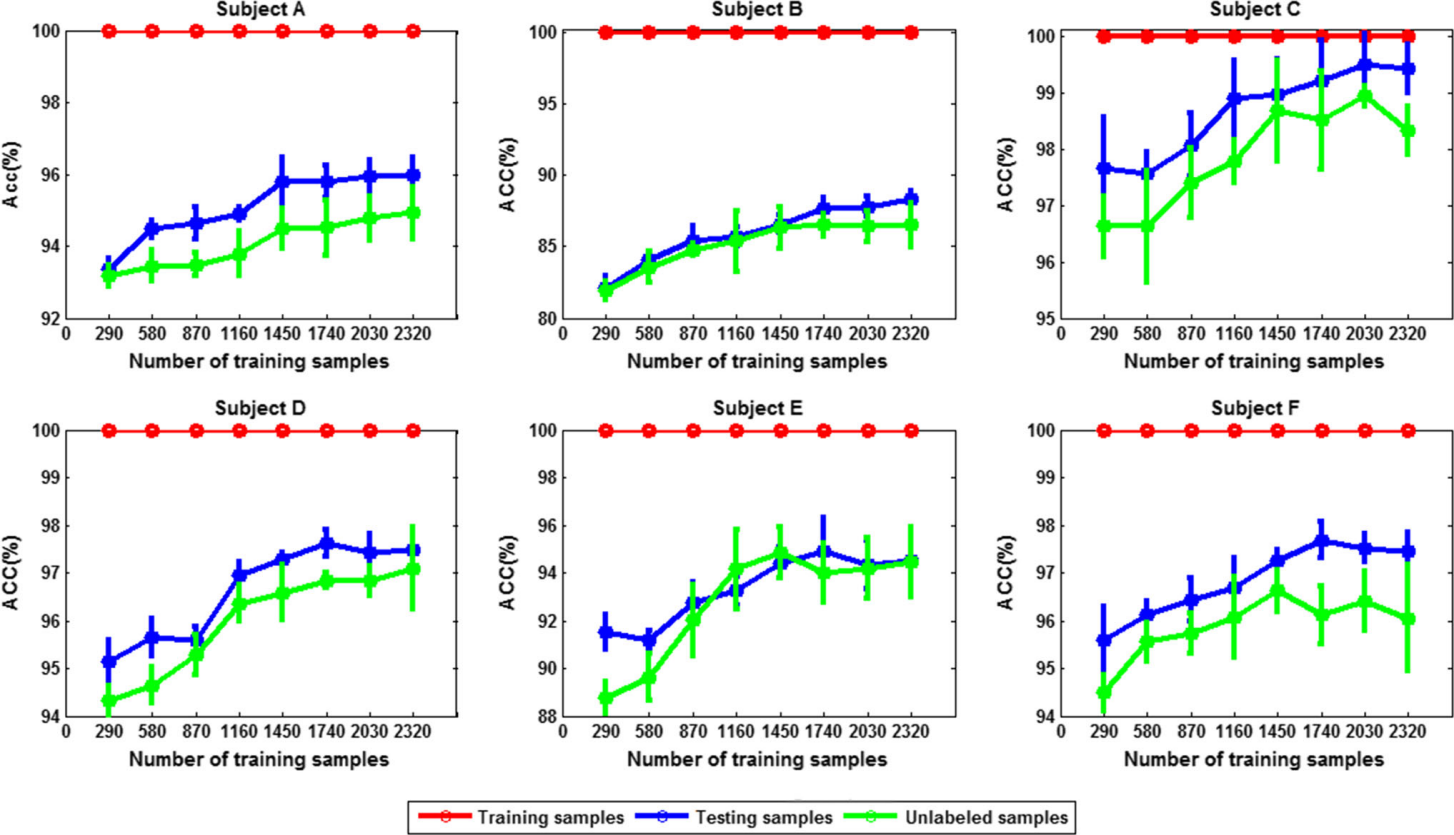
The classification accuracy versus size of unlabeled dataset for each subject (3-class case; size of labeled dataset: 29)

**Fig. 20:**
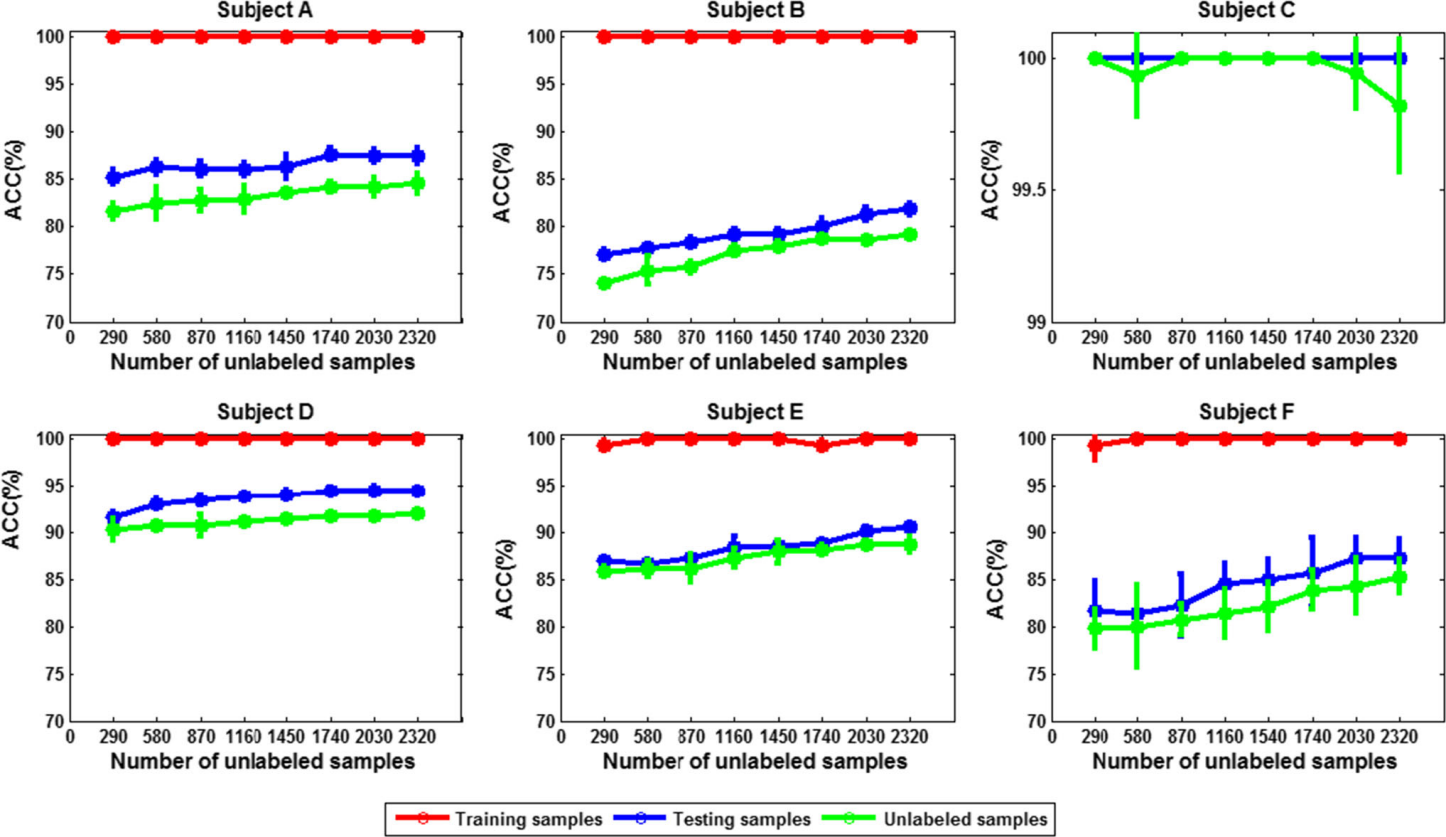
The classification accuracy versus size of unlabeled dataset for each subject (4-class case; size of labeled dataset: 29)

The corresponding classification accuracy is compared in Figs. 19 and 20 (mean ± SD). We can see that in either 3- or 4-class case, except for subject C, the classification accuracy for other five subjects is improved with the increase of the number of unlabeled data.

**Fig. 21:**
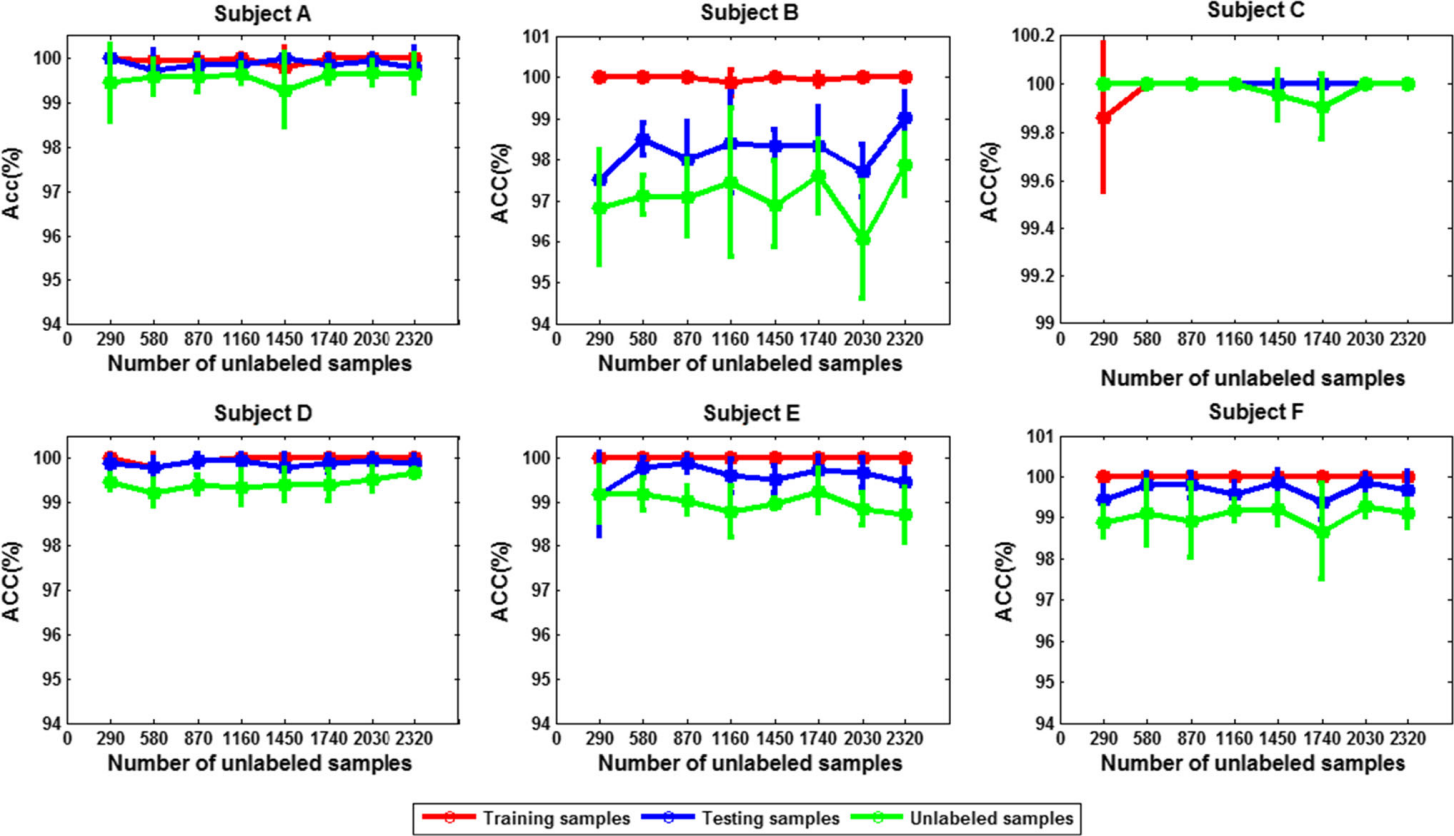
The subject average classification accuracy versus size of unlabeled dataset (3-class case; size of labeled dataset: 290)

**Fig. 22:**
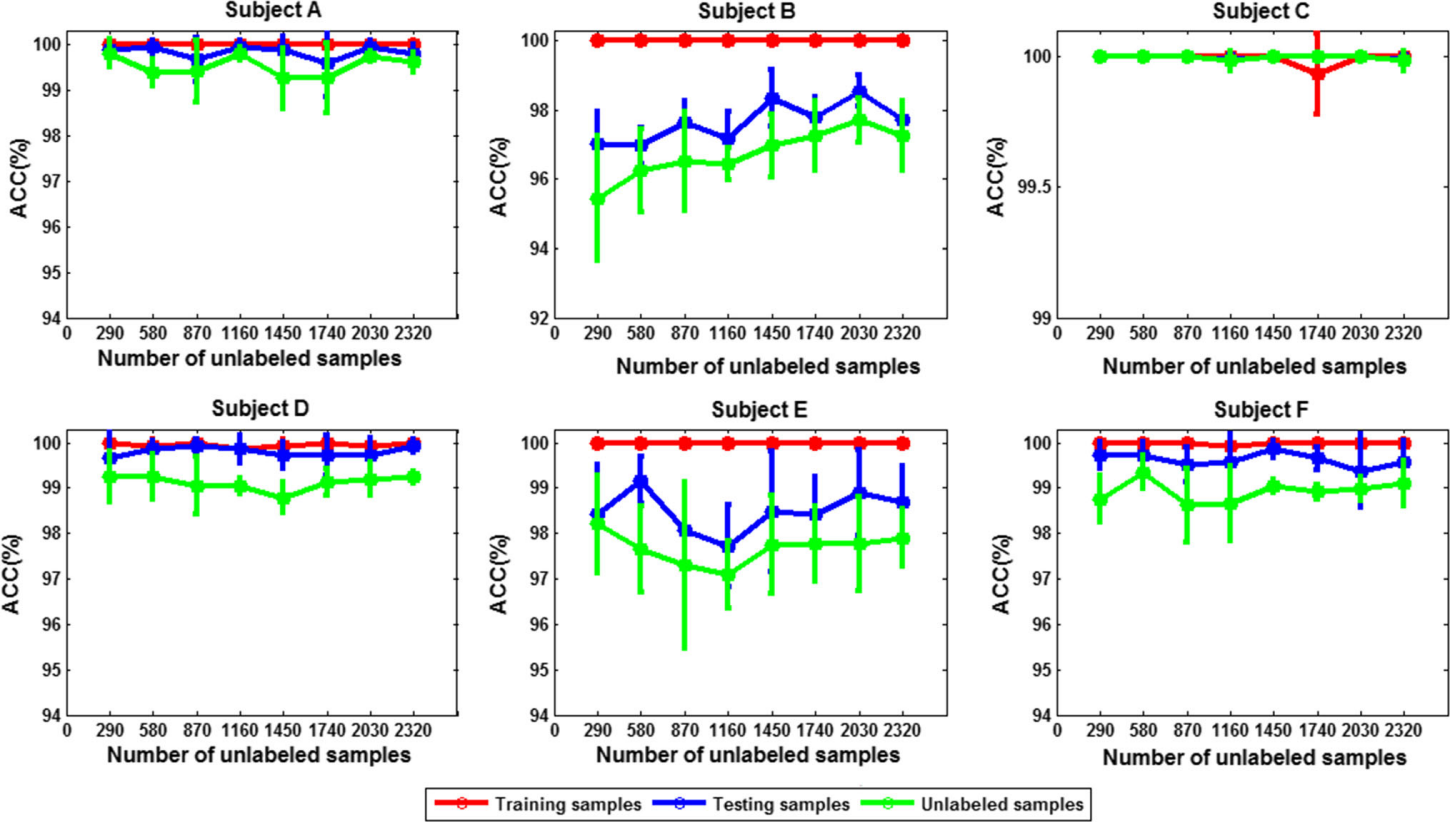
The subject average classification accuracy versus size of unlabeled dataset (4-class case; size of labeled dataset: 290)

Does this observation really indicate that the more unlabeled data, the better the classification performance? To answer this question, we gradually increase the number of the unlabeled data while fixing the size of the labeled set to 290. The corresponding classification accuracy for each subject is shown in Figs. 21 and 22. It can be seen that when the number of training samples is 290, increasing the number of unlabeled samples has little effect on the classification performance. Therefore, when the labeled data are sufficiently extensive to characterize the data manifold, increasing the unlabeled data does not have much effect on the performance improvement. The fundamental advantage of the SSL algorithm for risky MWL detection is that if the labeled set is smaller, it has outstanding advantages over supervised learning; conversely, if the labeled set is large, its performance is comparable to that of supervised learning algorithm.

#### Performance comparison between SSL and supervised learning

**Fig. 23:**
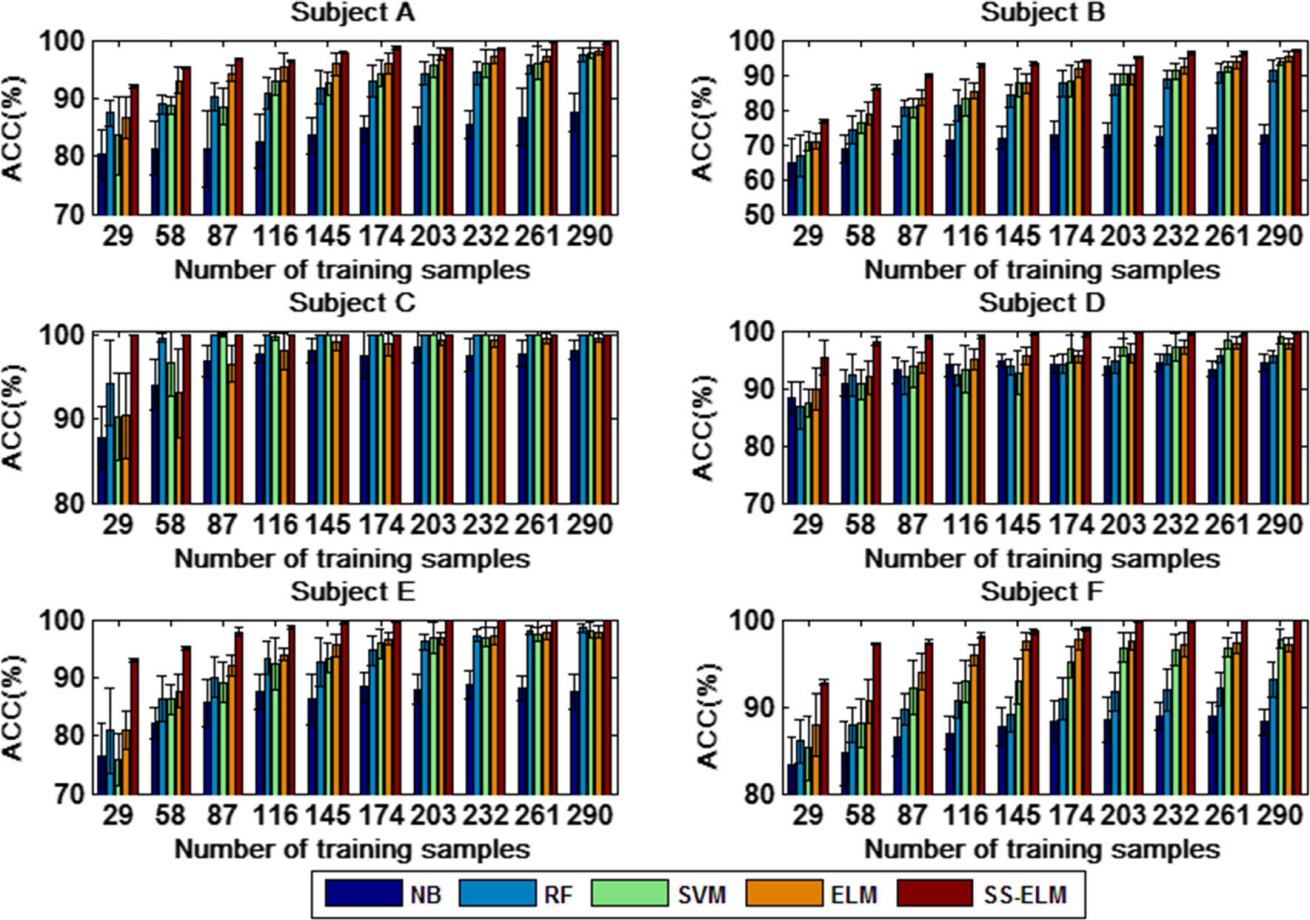
The classification accuracy of five different classifiers versus size of the labeled dataset (3-class case)

**Fig. 24:**
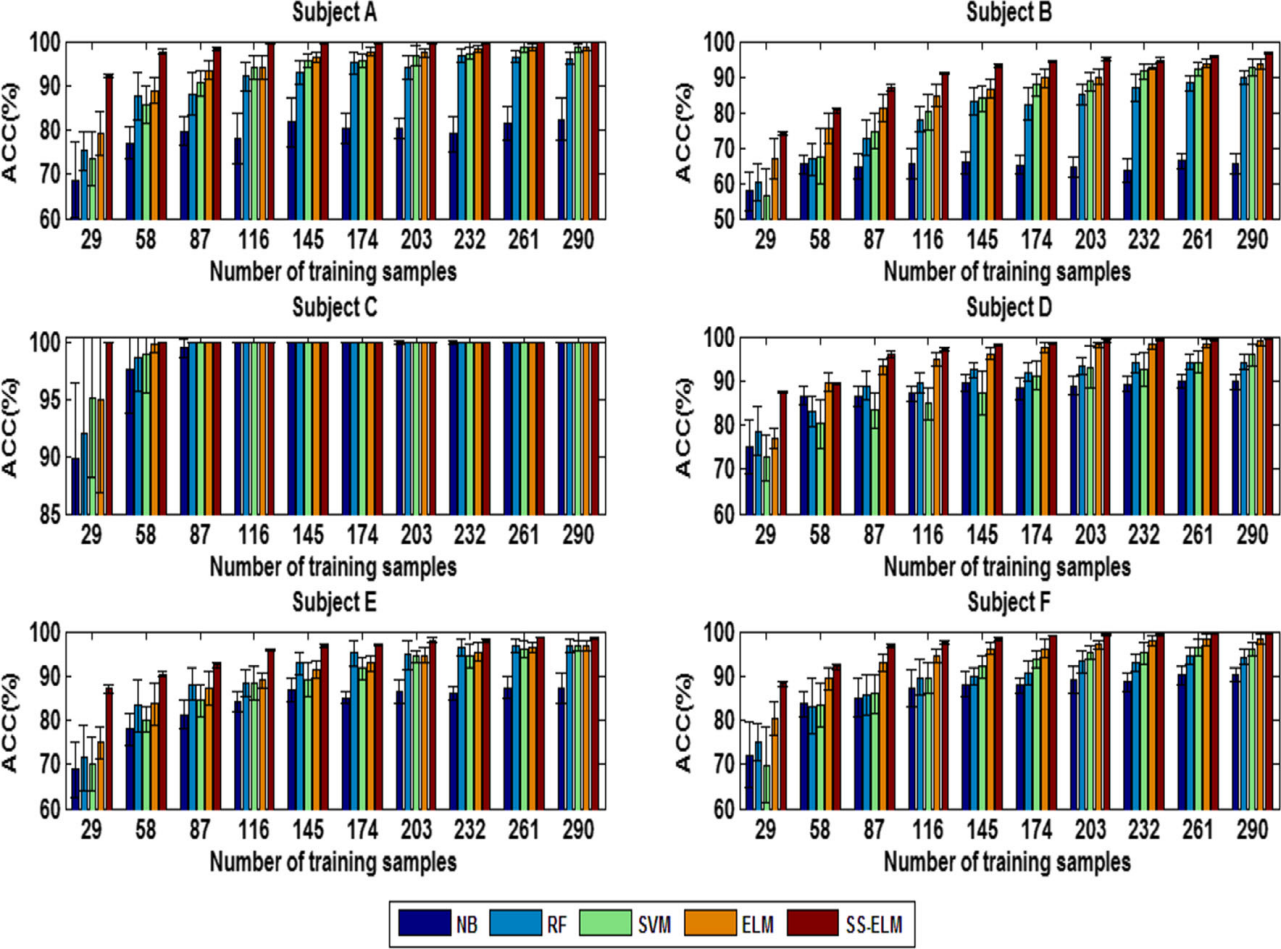
The classification accuracy of five different classifiers versus size of the labeled dataset (4-class case)

In order to show the potential of the SS-ELM method for MWL classification, we compare it with four classical supervised learning algorithms, namely Naive Bayesian (NB), Random Forest (RF), Support Vector Machines (SVM), and ELM. The comparative results are shown in Figs. 23 and 24 for 3- and 4-class problem, respectively.

Since the SSL algorithm takes full advantages of a large number of unlabeled data, its classification accuracy is shown to be superior to that of the four major supervising learning algorithms, but the improvement of accuracy depends on the size of training set. When the number of labeled data is small, the performance enhancement of the SS-ELM method is most significant compared with supervised learning algorithms. On the contrary, when the number of labeled samples is large, the difference in classification performance between them is only marginal. Consequently, the SSL algorithm would be more applicable to the special scenarios in which the labeled data is difficult or expensive to collect.

## Conclusion

Although ML techniques have shown promising performance in model-based MWL detection, a practical limitation of ML methods is the lack of a sufficient number of labeled data for modeling training. Labeling massive physiological data can be expensive or even erroneous, if not impossible, without enough domain-specific knowledge about OFS. As the SSL method only requires a small amount of labeled data, in the present investigation it is applied to real-time detection of high-risk MWL based on physiological data. We use SSL to make use of  the available unlabeled data with an aim to improve the accuracy of high-risk MWL detection.

The data analysis results obtained show that the proposed SSL approach is promising for risky MWL detection based on physiological signals. By taking advantage of the information contained in the unlabeled data, the graph-based SSL method can not only reduce the computational cost, but also improve the correct detection rate. With the increase of the number of the unlabeled data, even perfect classification can be fulfilled for certain datasets by using the SSL method. These findings suggest that by exploring the structure of the unlabeled data, we can gain and utilize additional information to improve the high-risk MWL detection performance.

## References

[CR1] Alves DK (2017). Real-time power measurement using the maximal overlap discrete wavelet-packet transform. IEEE Trans Ind Electron.

[CR2] Bobko N (1998). The mental performance of shiftworkers in nuclear and heat power plants of Ukraine. Int J Ind Ergonom.

[CR3] Cain B (2007). A review of the mental workload literature.

[CR4] Cannon J, Krokhmal PA, Chen Y, Murphey R (2012). Detection of temporal changes in psychophysiological data using statistical process control methods. Comput Methods Prog Biol.

[CR5] Chapelle O, Chi M, Zien A (2006) A continuation method for semi-supervised SVMs. In: Proceedings of the 23rd international. conference on machine learning (ICML’06), June 25–29, 2006, Pittsburgh, USA, pp 185–192

[CR6] Copson ET (1967). Asymptotic expansions.

[CR7] Dussault C, Jouanin J-C, Guezennec C-Y (2004). EEG and ECG changes during selected flight sequences. Aviat Space Environ Med.

[CR8] Dussault C (2005). EEG and ECG changes during simulator operation reflect mental workload and vigilance. Aviat Space Environ Med.

[CR9] Fournier LR, Wilson GF, Swain CR (1999). Electrophysiological, behavioral, and subjective indexes of workload when performing multiple tasks: manipulations of task difficulty and training. Int J Psychophysiol.

[CR10] Freeman FG (1999). Evaluation of an adaptive automation system using three EEG indices with a visual tracking task. Biol Psychol.

[CR11] Garla V, Taylor C, Brandt C (2013). Semi-supervised clinical text classification with Laplacian SVMs: an application to cancer case management. J Biomed Inform.

[CR12] Gevins A (1997). High-resolution EEG mapping of cortical activation related to working memory: effects of task difficulty, type of processing, and practice. Cereb Cortex.

[CR13] Gevins A (1998). Monitoring working memory load during computer-based tasks with EEG pattern recognition methods. Hum Factors.

[CR14] Gómez-Chova L, Camps-Valls G, Munoz-Mari J, Calpe J (2007) Semi-supervised cloud screening with Laplacian SVM. In: Proceedings of the 2007 IEEE international geoscience and remote sensing symposium (IGARSS 2007), 23–28 July 2007, Barcelona, Spain, pp 1521–1524

[CR15] Hankins TC, Wilson GF (1998). A comparison of heart rate, eye activity, EEG and subjective measures of pilot mental workload during flight. Aviat Space Environ Med.

[CR16] Hart SG, Staveland LE (1988). Development of NASA-TLX (Task Load Index): results of empirical and theoretical research. Adv Psychol.

[CR17] Hockey GRJ (1997). Compensatory control in the regulation of human performance under stress and high workload: a cognitive-energetical framework. Biol Psychol.

[CR18] Hockey GRJ (2003). Operator functional state: the assessment and prediction of human performance degradation in complex tasks.

[CR19] Hollender N (2010). Integrating cognitive load theory and concepts of human–computer interaction. Comput Hum Behav.

[CR20] Huang NE, Shen SSP (2014). Hilbert–Huang transform and its applications.

[CR21] Huang NE, Wu ZH (2008) A review on Hilbert–Huang transform: method and its applications to geophysical studies. Rev Geophys 46, RG2006, Paper No. 2007RG000228, pp 1–23

[CR22] Huang NE, Long SR, Shen Z (1996). The mechanism for frequency downshift in nonlinear wave evolution. Adv Appl Mech.

[CR23] Huang NE, Shen Z, Long SR (1998). The empirical mode decomposition and the Hilbert spectrum for nonlinear and nonstationary time series analysis. Proc R Soc Lond A.

[CR24] Huang G-B, Zhu Q-Y, Siew C-K (2006). Extreme learning machine: theory and applications. Neurocomputing.

[CR25] Huang G-B, Zhou H, Ding X, Zhang R (2012). Extreme learning machine for regression and multiclass classification. IEEE Trans Syst Man Cybern B Cybern.

[CR26] Huang G, Song S, Gupta JND, Wu C (2014). Semi-supervised and unsupervised extreme learning machines. IEEE Trans Cybern.

[CR27] Joachims T (1999) Transductive inference for text classification using support vector machines. In: Proceedings of the 16th international conference on machine learning (ICML1999), Morgan Kaufmann, June 27–30, 1999, Bled, Slovenia, pp 200–209

[CR28] Krishna PKM, Ramaswamy K (2017). Single channel speech separation based on empirical mode decomposition and Hilbert Transform. IET Signal Proc.

[CR29] Lal SKL, Craig A (2001). A critical review of the psychophysiology of driver fatigue. Biol Psychol.

[CR30] Lamti HA, Ben Khelifa MM, Hugel V (2019). Mental fatigue level detection based on event related and visual evoked potentials features fusion in virtual indoor environment. Cogn Neurodyn.

[CR31] Mahfouf M, Zhang J, Linkens DA et al (2007) Adaptive fuzzy approaches to modelling operator functional states in a human–machine process control system. In: Proceedings of the IEEE international conference on fuzzy systems (FUZZ-IEEE 2007), 23–26 July 2007, London, UK, pp 1–6

[CR32] McClosky D, Charniak E, Johnson M (2006) Effective self-training for parsing. In: Proceedings of the conference on human language technology of the North American chapter of the ACL, pp 152–159

[CR33] Mora-Sánchez A, Pulini A, Gaume A (2020). A brain–computer interface for the continuous, real-time monitoring of working memory load in real-world environments. Cogn Neurodyn.

[CR34] Okamoto M (2004). Three-dimensional probabilistic anatomical cranio-cerebral correlation via the international 10–20 system oriented for transcranial functional brain mapping. Neuroimage.

[CR35] Swangnetr M, Kaber DB (2013). Emotional state classification in patient–robot interaction using wavelet analysis and statistics-based feature selection. IEEE Trans Hum–Mach Syst.

[CR36] Wang Y, Zhang J, Wang R (2016) Mental workload recognition by combining wavelet packet transform and kernel spectral regression techniques. In: Proceedings of 13th IFAC symposium on analysis, design, and evaluation of human–machine systems (HMS2016), Kyoto, Japan, Aug. 30–Sep. 02, 2016; IFAC-PapersOnLine, vol 49.19, pp 561–566

[CR37] Wang H, Dragomir A, Abbasi NI (2018). A novel real-time driving fatigue detection system based on wireless dry EEG. Cogn Neurodyn.

[CR38] Wilson GF, Fisher F (1991). The use of cardiac and eye blink measures to determine flight segment in F4 crews. Aviat Space Environ Med.

[CR39] Wilson GF, Russell CA (1999) Operator functional state classification using neural networks with combined physiological and performance features. In: Proceedings of the human factors and ergonomics society annual meeting, vol 43. No. 20, Sage, Los Angeles, CA

[CR40] Wilson GF, Russell CA (2003). Operator functional state classification using multiple psychophysiological features in an air traffic control task. Hum Factors.

[CR41] Wilson GF, Russell CA (2003). Real-time assessment of mental workload using psychophysiological measures and artificial neural networks. Hum Factors.

[CR42] Wilson GF, Russell CA (2007). Performance enhancement in an uninhabited air vehicle task using psychophysiologically determined adaptive aiding. Hum Factors.

[CR43] Yin Z, Zhang J (2014). Operator functional state classification using least-square support vector machine based recursive feature elimination technique. Comput Methods Programs Biomed.

[CR44] Yin Z, Zhang J (2014). Identification of temporal variations in mental workload using locally-linear-embedding-based EEG feature reduction and support-vector-machine-based clustering and classification techniques. Comput Methods Programs Biomed.

[CR49] Zeng H, Yang C, Dai G (2018). EEG classification of driver mental states by deep learning. Cogn Neurodyn.

[CR45] Zhang J, Wang X, Mahfouf M, Linkens DA (2008) Use of heart rate variability analysis for quantitatively assessing operator’s mental workload. In: Proceedings of international conference on biomedical engineering and informatics (BMEI 2008), Sanya, China, 27–30 May 2008, pp 668–672

[CR46] Zhang J, Liu H, Peng X, Raisch J, Wang R (2013). Classifying human operator functional state based on electrophysiological and performance measures and fuzzy clustering method. Cogn Neurodyn.

[CR47] Zhang J, Qin P, Raisch J, Wang R (2013). Predictive modeling of human operator cognitive state via sparse and robust support vector machines. Cogn Neurodyn.

[CR48] Zhang J, Yang S, Wang R (2016). Operator functional state estimation based on EEG-data-driven fuzzy model. Cogn Neurodyn.

[CR50] Zhu X, Sammut C, Webb GI (2017). Semi-supervised learning. Encyclopedia of machine learning and data mining.

